# Fenbendazole acts as a moderate microtubule destabilizing agent and causes cancer cell death by modulating multiple cellular pathways

**DOI:** 10.1038/s41598-018-30158-6

**Published:** 2018-08-09

**Authors:** Nilambra Dogra, Ashok Kumar, Tapas Mukhopadhyay

**Affiliations:** 1 National Centre for Human Genome Studies and Research, Panjab University, Sector-14, Chandigarh, 160014 India; 2 Present Address: Department of Experimental Medicine and Biotechnology, Postgraduate Institute of Medical Education and Research, Sector-12, Chandigarh, 160012 India; 3 Present Address: Centre for Systems Biology and Bioinformatics, Panjab University, Sector-25, Chandigarh, 160014 India

## Abstract

Drugs that are already clinically approved or experimentally tested for conditions other than cancer, but are found to possess previously unrecognized cytotoxicity towards malignant cells, may serve as fitting anti-cancer candidates. Methyl N-(6-phenylsulfanyl-1H benzimidazol-2-yl) carbamate [Fenbendazole, FZ], a benzimidazole compound, is a safe and inexpensive anthelmintic drug possessing an efficient anti-proliferative activity. In our earlier work, we reported a potent growth-inhibitory activity of FZ caused partially by impairment of proteasomal function. Here, we show that FZ demonstrates moderate affinity for mammalian tubulin and exerts cytotoxicity to human cancer cells at micromolar concentrations. Simultaneously, it caused mitochondrial translocation of p53 and effectively inhibited glucose uptake, expression of *GLUT* transporters as well as hexokinase (*HK II*) - a key glycolytic enzyme that most cancer cells thrive on. It blocked the growth of human xenografts in *nu/nu* mice model when mice were fed with the drug orally. The results, in conjunction with our earlier data, suggest that FZ is a new microtubule interfering agent that displays anti-neoplastic activity and may be evaluated as a potential therapeutic agent because of its effect on multiple cellular pathways leading to effective elimination of cancer cells.

## Introduction

The importance of microtubules in cell division, motility, intracellular trafficking and their role in modulating cellular shape according to the environment has made them one of the most successful targets of anticancer therapy. Agents that perturb the microtubule dynamics have been widely used in cancer treatment^1–4^. Considering the relative success of mitotic agents in the treatment of cancer, microtubules may be termed as one of the best cancer targets identified till now^5^.

Microtubule targeting agents can be broadly classified into two major classes. The first class consists of microtubule-destabilizing agents, which inhibit microtubule polymerization. This class of anti-mitotic drugs includes several compounds such as the vinca alkaloids (vinblastine, vincristine, vinorelbine, vindesine, vinflunine), estramustine, colchicine and combretastatins, that are being used clinically or are under clinical investigation for cancer treatment. The second class is comprised of microtubule-stabilizing agents. These agents include paclitaxel, docetaxel, epothilones, and discodermolide^6^. The consequence of disrupting tubulin and microtubule dynamics with both these classes of drugs in dividing cells is metaphase arrest and induction of apoptosis.

Fenbendazole (methyl *N*-(6-phenylsulfanyl-1H-benzimidazol-2-yl) carbamate) is a broad-spectrum benzimidazole anthelminthic approved for use in numerous animal species^7^. Repurposing of veterinary drugs showing promising results for human use can result in considerable time and cost reduction required to develop new drugs. Fenbendazole is known to have a high safety margin and most species tolerate it very well. It has very low degree of toxicity and high degree of safety in experimental animals^8–12^. In this study, we show that fenbendazole (FZ) exhibits a moderate microtubule depolymerizing activity towards human cancer cells, but possesses a potent antitumor effect as evident from *in vitro* and *in vivo* experiments. Our results indicate that FZ exerts its antitumor effect through the disruption of microtubule dynamics, p53 activation and the modulation of genes involved in multiple cellular pathways. FZ treatment also resulted in reduced glucose uptake in cancer cells due to down regulation of *GLUT* transporters and key glycolytic enzymes.

Since the process of tumorigenesis involves a number of genes and proteins altering various cell signaling pathways, single-target drugs show limited efficacy and may lead to drug resistance^13–15^. Agents having multiple cellular targets, therefore, are expected to have improved efficacy besides the ability to circumvent the likelihood of developing resistance.

Overall, the present work demonstrates a pleiotropic effect of FZ on cancer cells leading to cell death. Thus, FZ may have a potential therapeutic application.

## Results

### FZ destabilizes tubulin network in human NSCLC cells

**Figure 1:**
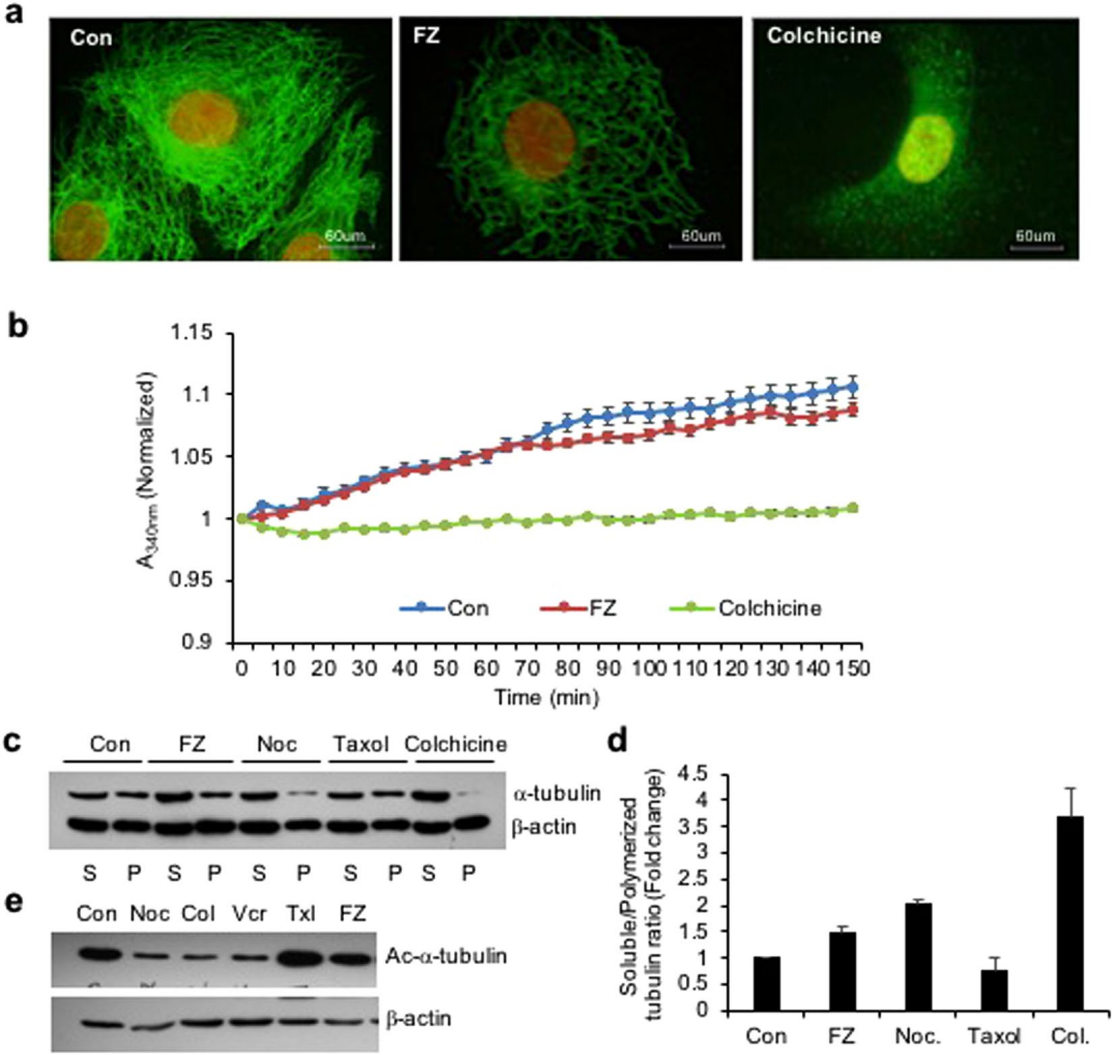
FZ treatment alters tubulin network of human cancer cells. (**a**) A549 cells were treated with 1 uM FZ or 50 ng/ml colchicine for 24 h. Following treatment, the cells were processed for immunofluorescence using anti α-tubulin primary and FITC conjugated secondary antibodies. (Nuclei were counter stained with propidium iodide) (**b**) bovine tubulin (1.8 mg/mL) was incubated with DMSO (control), FZ (10 uM) or colchicine (100 nM) and the effect on polymerization was monitored spectrophotometrically by measuring turbidity at 340 nm as described under “Methods.” (**c**) Cells were treated with FZ, nocodazole, taxol or colchicine for 24 h and then lysed and fractionated into soluble (S) and polymerized (P) extracts. The extracts were separated with SDS-PAGE, transferred onto PVDF membranes and probed with both anti-α-tubulin and anti-β-actin antibodies. A representative immunoblot analysis in A549 cells is shown. (**d**) Intensity of each band of the immunoblot was measured by the NIH ImageJ program, and the ratios of soluble and polymerized tubulin and β-actin in each treatment were calculated. (**e**) Cells were treated with different MTAs as indicated for 24 h and western blotting was then performed using Ac-α-tubulin (6–11B-1) specific and β-actin antibodies. (Full-length uncropped blots are included in Supplementary Fig. S6).

Benzimidazole carbamates have been reported to inhibit tubulin polymerization and disrupt microtubule function in parasite cells^16,17^. Results from *in vitro* studies using enriched extracts of helminthic and mammalian tubulin have suggested that tubulin is the primary molecular target of the benzimidazoles^18^. Therefore, to examine the effect of FZ on mammalian microtubule network organization, human non small cell lung carcinoma (NSCLC) A549 cells were treated with 1 uM FZ for 24 h and processed for immunofluorescence using α tubulin antibody. Colchicine was used as a positive control. Results showed that FZ treatment caused a partial alteration of the microtubule network (Fig. 1a). The microtubule cage around the nucleus appeared to have lost its intactness when compared with the control mock treated cells. However, this modification in the organization was not as marked as in case of colchicine treatment, which showed complete depolymerization of microtubules into tubulin subunits. This data suggests that FZ causes distorted microtubule framework of the cells.

The effect of FZ on tubulin polymerization was further evaluated by an *in vitro* assay. Purified bovine tubulin was incubated with FZ, and tubulin polymerization was recorded over time. The results showed mild inhibition of tubulin polymerization by FZ *in vitro* which was not as pronounced as in case of colchicine treatment. (Fig. 1b)

Next, the effect of FZ on tubulin polymerization was compared with that of other microtubule destabilizing agents like nocodazole and colchicine. Polymerized and soluble fractions were prepared after 24 h drug treatment and western blot was performed using α-tubulin and β-actin antibodies (Fig. 1c). Tubulin bands of polymerized and soluble fractions were quantified after normalization with their respective β-actin bands which served as an internal control (Fig. 1d). There was a modest decrease in polymeric tubulin in FZ treated cells as compared to control untreated cells, whereas polymerized form of tubulin was nearly absent in colchicine treated cells. The result confirms the relatively mild tubulin depolymerising activity of FZ as compared to other known microtubule disrupting agents like nocodazole and colchicine.

A major limiting factor of taxanes and vinca alkaloids is their dose-limiting toxicity and susceptibility to multidrug drug-resistance (MDR) occurring commonly due to the high expression of p-glycoprotein (p-gp; MDR1)^19,20^. Overexpression of β-tubulin isoforms and mutations are also known to confer resistance to taxanes^21^. Unlike taxanes and vinca alkaloids, agents targeting the colchicine-binding site are advantageous in that they show minimal multidrug resistance in addition to their ability to overcome the effect of β-tubulin isoforms’ overexpression^22–24^. However, the major drawback of colchicine and its derivatives is their acute toxicity to humans^22,25^. Therefore, a microtubule inhibitor that binds to the colchicine-binding site but has low toxicity can be highly efficacious^26,27^. The result of a fluorescence based competitive colchicine binding assay suggests that FZ may bind to the tubulin at the colchicine binding site (Fig. S1).

Tubulin acetylation has been associated with the stability of microtubules. Therefore, to examine the acetylation status of tubulin following treatment, human NSCLC cells were treated with different microtubule targeting agents for 24 h and the cell extracts were subjected to western blot analysis using Ac-α-tubulin specific antibody (6–11B-1). As shown in Fig. 1e, while nocodazole, colchicine and vincristine resulted in a marked reduction of acetylated tubulin, FZ did not alter the amount of acetylated tubulin as compared with control mock treated cells. This result further confirmed the relatively mild effect of FZ on mammalian tubulin as compared with other known microtubule depolymerizing agents.

### FZ is not a P-gp substrate or inhibitor

Development of drug resistance is a major concern in cancer treatment. Multidrug resistance (MDR) caused by the overexpression of the MDR-1 gene that encodes P-glycoprotein (P-gp) is a critical mechanism of drug resistance which results in a cross-resistance to multiple classes of drugs^28,29^. A large number of commonly used chemotherapy drugs like taxanes and vinca alkaloids are P-gp substrates^30^. However, efforts to inhibit P-gp have not shown encouraging results due to unavoidable side effects^31,32^. Therefore, discovery and development of novel anti-proliferative compounds that are not substrates of P-gp is an effective approach to overcome drug resistance.

**Figure 2:**
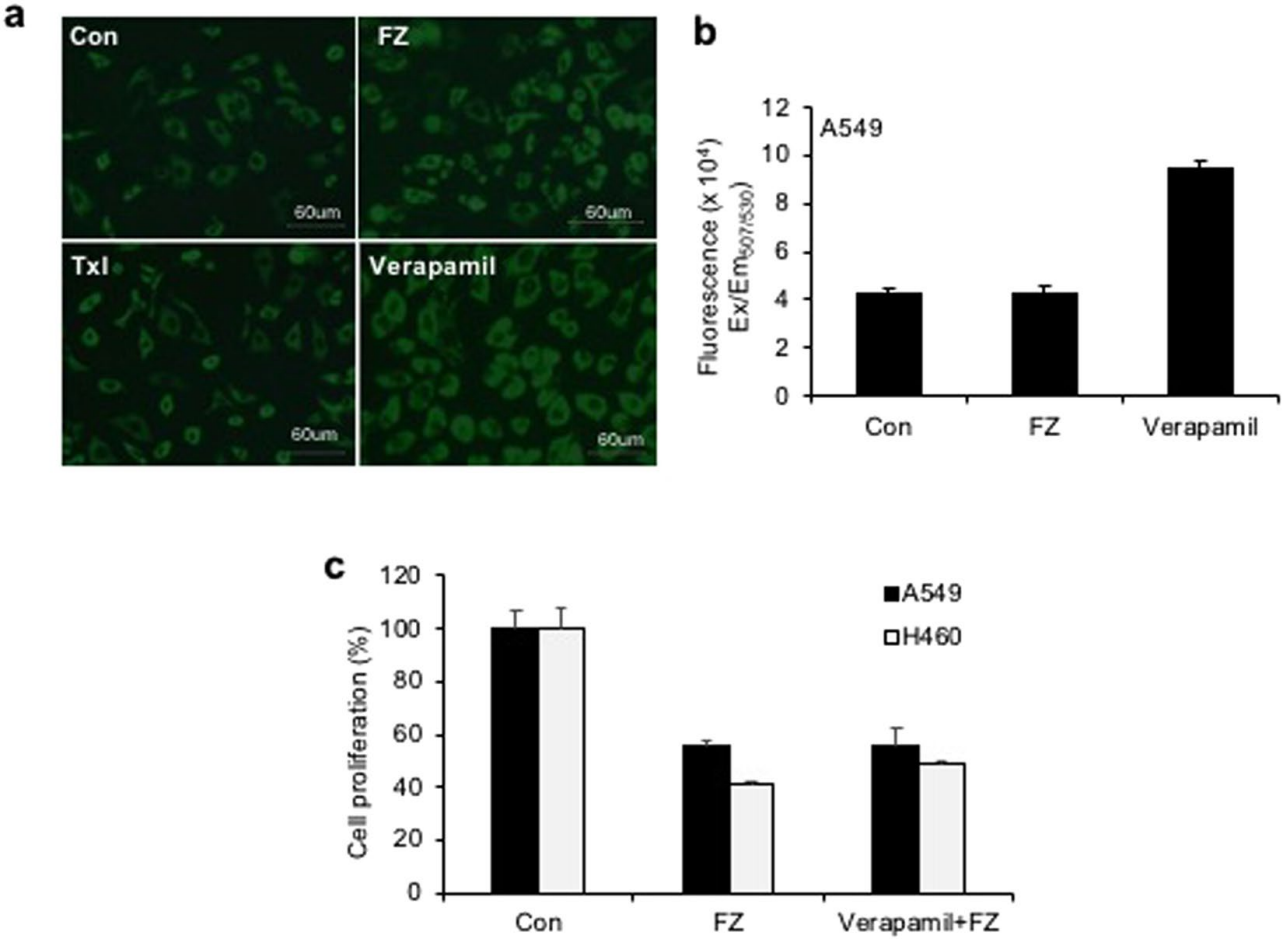
P-gp inhibition has no effect on FZ mediated cell death. (**a**) A549 cells were left untreated or treated with 10 uM FZ or 10 uM Verapamil for 6 h. Rh123 was then added and fluorescence images were acquired after washings with PBS as described under “Methods”. (**b**) The cells were treated as before and the fluorescence was measured at Ex_507_/Em_529_ using a Tecan multimode plate reader. (**c**) A549 and H460 cells were treated with 1 uM FZ in the absence or presence of 10 uM verapamil for 24 h. Cell proliferation was then measured by MTT assay.

To test whether FZ is a substrate or inhibitor of P-gp, we investigated cancer cell growth inhibition by FZ in the presence of P-gp inhibitor verapamil. The results showed that inhibition of P-gp by verapamil did not enhance the inhibitory effect of FZ on cancer cell proliferation (Fig. 2c). The fluorescent dye rhodamine 123 (Rho123) is a well-known reference P-gp substrate frequently used to determine the P-gp inhibitory potential of drugs^33^. No significant difference in Rho123 accumulation was observed between control untreated and FZ treated cells, implying the absence of any interaction of FZ with P-gp. (Fig. 2a,b) In the presence of verapamil, the treated and untreated cells showed comparable levels of Rho123 accumulation affirming that FZ is not a substrate or inhibitor of P-gp.

### FZ treatment results in early G2/M block accompanied by cell death

**Figure 3:**
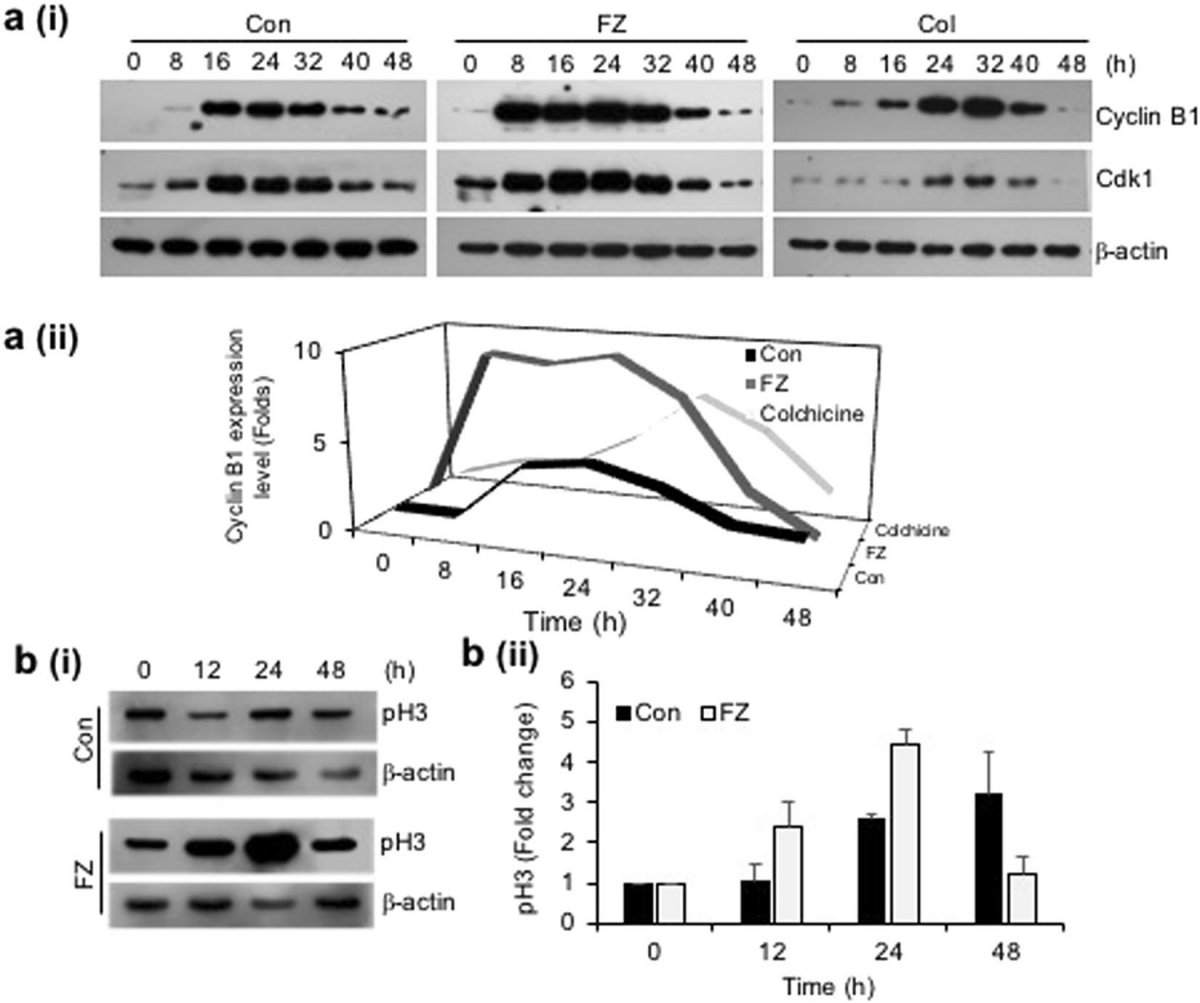
FZ causes early elevation of cyclin B1 levels and induces mitotic arrest. **a(i) & a(ii)** A549 cells were synchronized by serum starvation for 48 h and then left untreated or treated with 1 uM FZ or 50 nM colchicine for the indicated time intervals. The cell extracts were then processed for western immunoblotting using cyclin B1, cdk1 and β-actin antibodies. **b(i) & b(ii)** A549 cells were treated with 1 uM FZ for the indicated time intervals and the extracts were then processed for western blotting using pH3 and β-actin antibodies. The bands were quantitated using ImageJ software. (Full-length uncropped blots are included in Supplementary Fig. S6).

**Figure 4:**
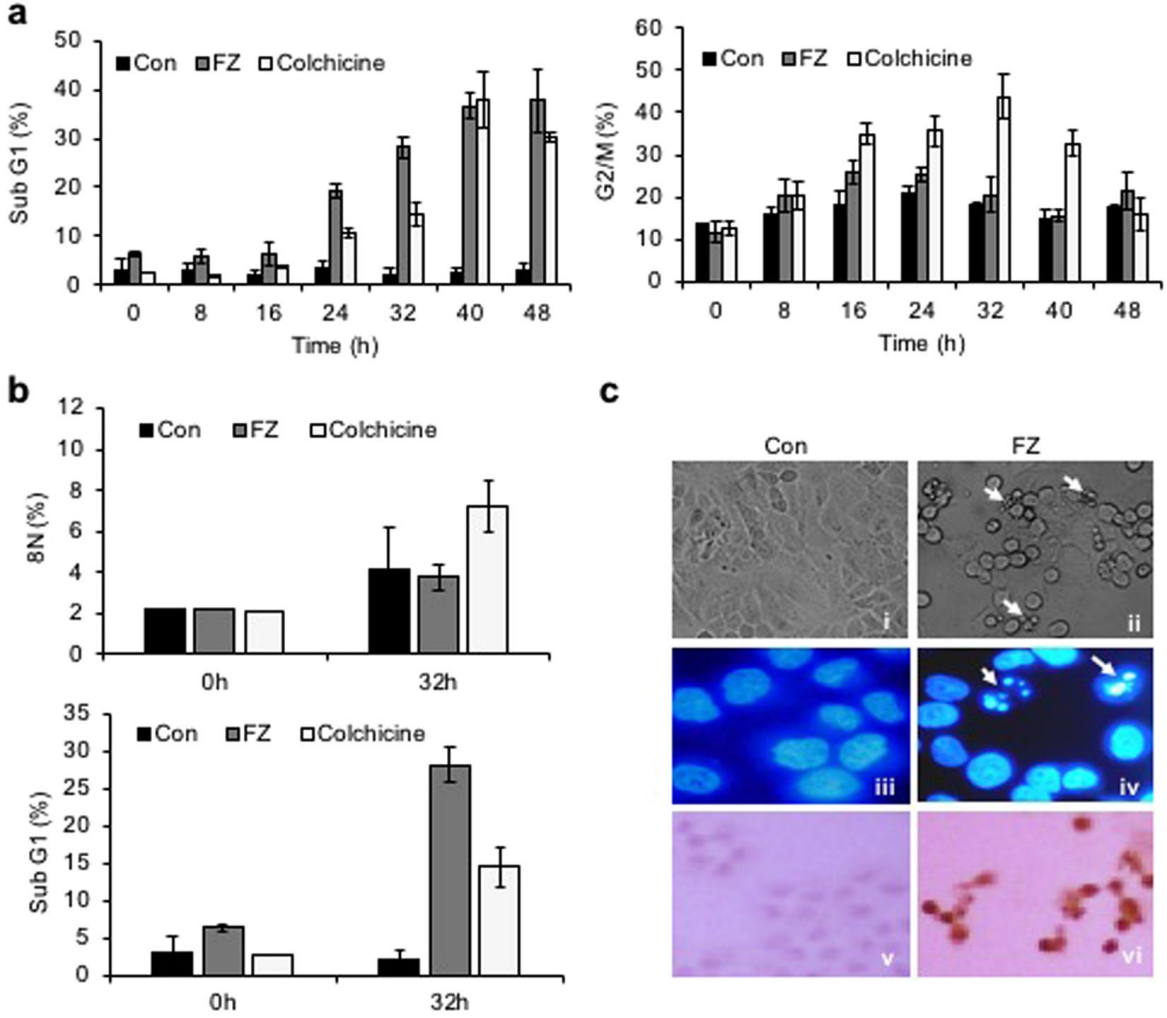
Mitotic arrest followed by cell death in response to FZ in human NSCLC cells. (**a** and **b**) Cells were treated after synchronization as before and FACS analysis was done to detect the fraction of populations in different phases of cell cycle, as well as apoptotic cells. (**c**) H460 cells were treated with 1 uM FZ for 24 h and cell morphology was observed under phase contrast microscope (*i & ii*) or after Hoechst 33342 staining under fluorescence microscope (*iii & iv*). TdT staining was done to detect apoptotic nuclei (*v & vi*).

Since inhibition of tubulin polymerization blocks cell-cycle progression and may induce mitotic catastrophe, the effect of fenbendazole on the cell cycle progression was examined. A549 cells were synchronized by serum starvation for 48 h and treated with 1 uM FZ for different time intervals. During cell division, cyclin B1 binds to cyclin-dependent kinase 1 (CDK1) allowing the transition from G2 to mitosis. Thereafter, progression from metaphase to anaphase requires ubiquitination and proteasome mediated degradation of cyclin B1 induced by anaphase-promoting complex (APC)^34^. Our results show an early elevation of cyclin B1/CDK1 levels in FZ treated cells (8 h as compared to 16 h in case of control untreated cells) (Fig. 3a). In addition, histone pH3 (Ser10), an indicator of mitotic progression, was used to evaluate whether FZ induced cell cycle arrest specifically in the mitosis phase. As seen in Fig. 3b, p-histone H3 (Ser10) was found to be up-regulated at 12 and 24 h post FZ treatment in A549 cells. This data confirms that FZ causes cell cycle arrest in the mitotic phase in human NSCLC cells. Cells in different phases of cell cycle and those undergoing apoptosis were quantified by flow cytometry. The number of apoptotic cells increased in a time dependent manner with simultaneous decrease in cyclin B1 levels, and ~30% cells had undergone apoptosis after 32 h of FZ treatment (Fig. 4a). The cyclin B1 levels went down further at 40 and 48 h of treatment, corresponding to a further increase in the percentage of apoptotic cells. Thus, a decrease in cyclin B1 levels, with a concomitant increase in apoptotic cell death suggests that A549 cells apparently underwent mitotic exit followed by cell death in response to FZ treatment.

While a significant apoptotic population (sub-G_1_ phase) was observed after 32 h FZ treatment, in contrast, colchicine treated cells underwent polyploidy at the same time point (Fig. 4b). FZ enforced cells to accumulate in mitosis, leading to a dramatic increase in apoptosis without evidence of progression to a G1-like status. Figure 4c shows apoptotic cells following 24 h FZ treatment. Overall, this data suggests that FZ causes cell-cycle arrest and mitotic cell death, consistent with its effects as a microtubule inhibitor. In our earlier work, we reported the absence of any effect of broad spectrum caspase inhibitor Z-VAD-FMK on FZ induced cell death^35^. The present data suggesting mitotic cell death caused by FZ offers a possible explanation for this result since mitotic cell death can be caspase independent and is known to remain unaffected by the caspase inhibitor Z-VAD-FMK^36,37^.

### Tumour cell lines with wild-type p53 show enhanced sensitivity to FZ induced apoptosis

**Figure 5:**
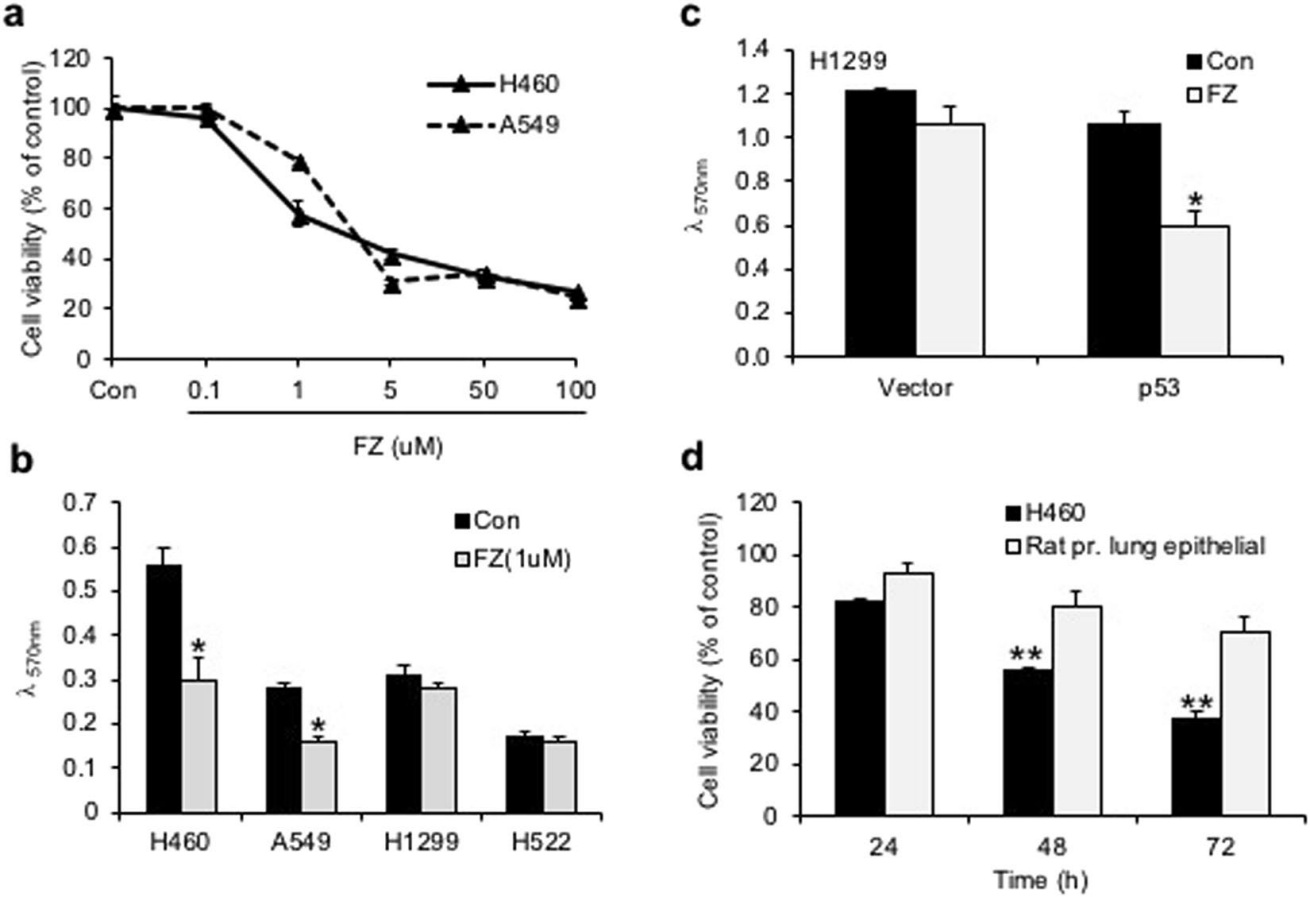
FZ mediated inhibition of cancer cells *in vitro* is affected by p53 status. (**a**) Human H460 or A549 cells were plated onto 96-well tissue culture plates. Cells were left untreated or treated with different doses of FZ for 48 h. Cell viability was measured by MTT assay. (**b**) H460, A549, H522 or H1299 cells were treated with 1 uM FZ for 48 h and cell viability was measured by MTT assay. (**c**) H1299 cells were transfected with p53 expression construct or with vector alone. After 16 h of transfection, cells were left untreated or treated with FZ for 24 h following which cell viability was measured by MTT assay. (**d**) H460 cells or primary lung epithelial cells derived from rat lung tissue were treated with 1 uM FZ for different time points as indicated. Cell viability was determined by MTT assay. The significance level was set at *p* < 0.05. (*p < 0.05)

**Figure 6:**
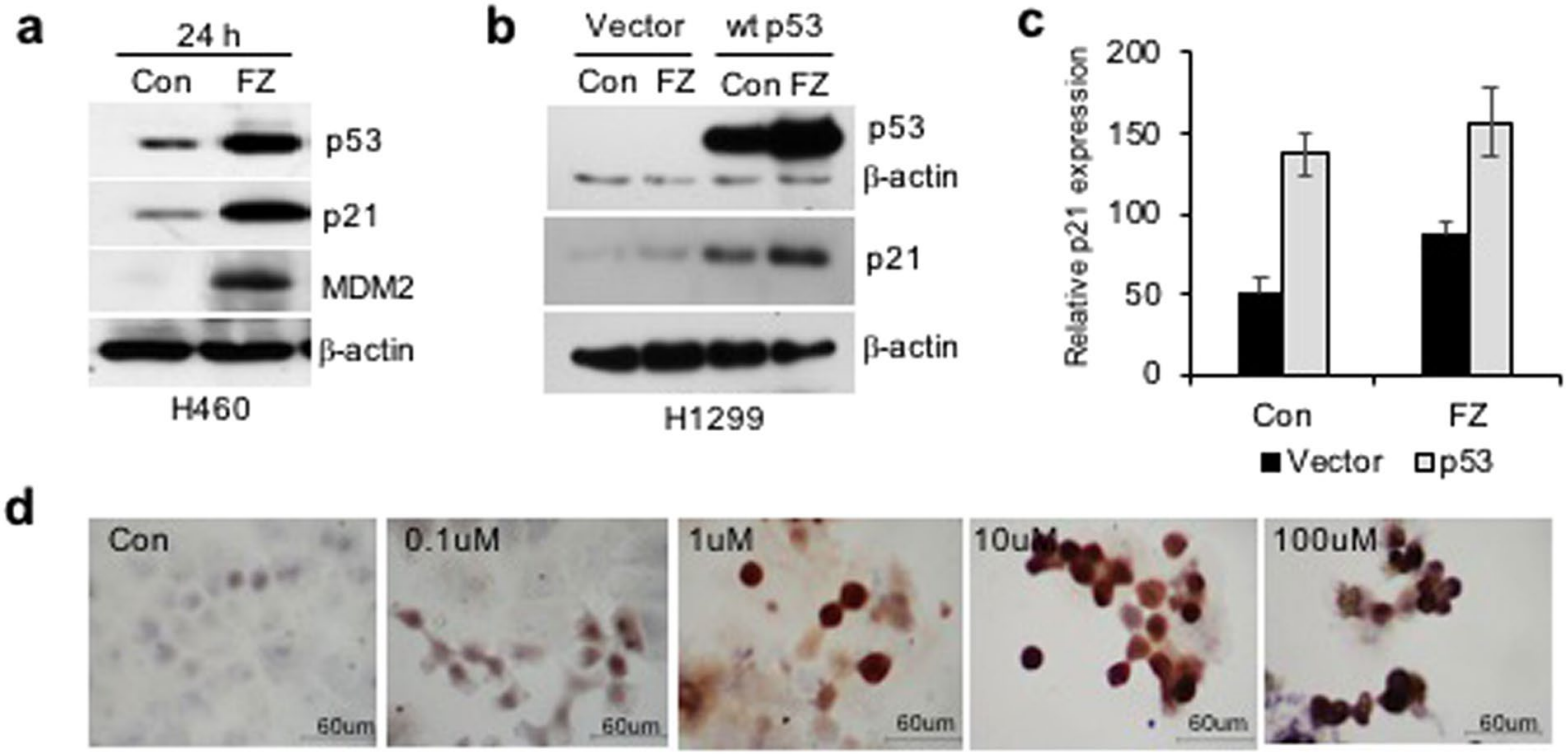
Apoptosis and p53 induction in human NSCLC cell lines following FZ treatment. (**a**) Western blot was performed for p53 target genes after 24 h of FZ treatment in H460 cells. (**b**) H1299 cells were transiently transfected with p53 expression construct and left untreated or treated with FZ for 24 h. Western blot was then done for p53 and p21. (**c**) Quantitation of “b” using Image J software. (Full-length uncropped blots are included in Supplementary Fig. S6) (**d**) immunostaining for p53 in H460 cells after treatment with increasing concentrations of FZ for 24 h.

Treatment of H460 and A549 human NSCLC cell lines with 1 uM FZ significantly reduced cell growth as determined by MTT assay (Fig. 5a). Tumour cell lines having wild-type (WT) p53 appeared to be highly sensitive to FZ action as compared to p53 mutant or null cells (Fig. 5b), indicating enhanced apoptosis inducing activity of FZ in the presence of WT p53. Consistent with this data, p53 null H1299 cells exposed to FZ following transient transfection with WT p53 construct showed enhanced apoptotic cell death (Fig. 5c). Remarkably, FZ showed less toxicity towards primary epithelial cells cultured from rat lung tissue as compared to a lung cancer cell line (Fig. 5d). Wild-type p53 target genes were induced upon FZ treatment (Fig. 6a), suggesting that FZ induced WT p53 protein is transcriptionally active. However, FZ could also induce p21 independent of p53 (Fig. 6b,c), which may be via its effect on the proteasomal pathway as reported earlier^35^. Dose dependent FZ treatment led to increased nuclear accumulation of WT p53 in these cells which correlated well with enhanced apoptotic activity (Fig. 6d). Tubulin acetylation has been reported to enhance stress-mediated translocation of p53 to the nucleus where it is transcriptionally active^38,39^. Hence, enhanced p53 nuclear accumulation is concurrent with earlier data showing steady levels of acetylated tubulin in FZ treated cells. Altogether, evidence supports a proactive role of transcriptionally active p53 in augmenting cell death following mild microtubule disruption by FZ.

### FZ induces p53 mitochondrial translocation

**Figure 7:**
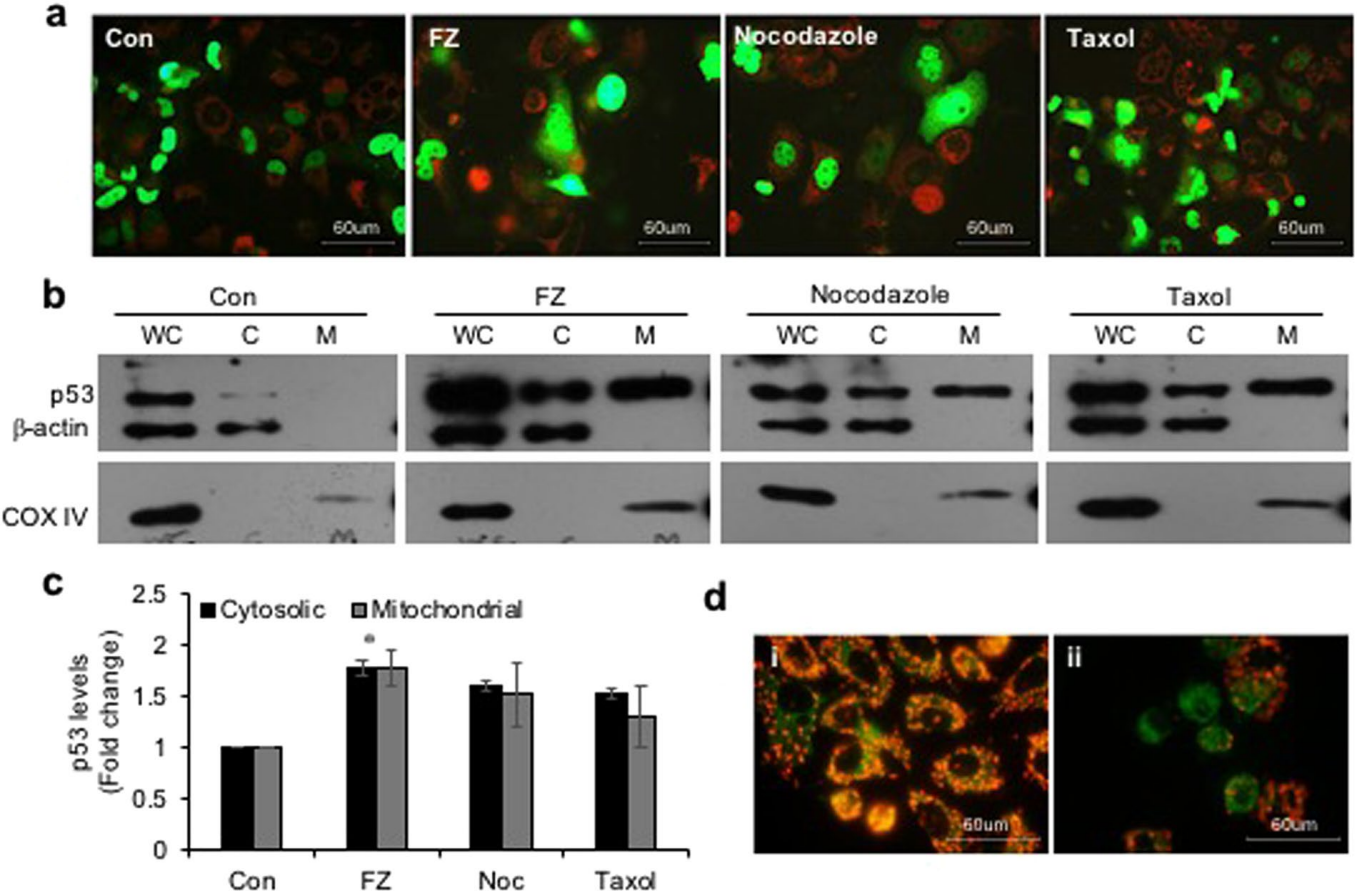
FZ treatment results in increased p53 translocation to mitochondria. (**a**) H1299 cells were transiently transfected with GFP-p53 expression construct and treated as before. They were then stained with red mitotracker dye (Molecular Probes) and fluorescent images were acquired using a Nikon fluorescence microscope. (**b**) H460 cells were treated with 1 uM FZ, 500 nM nocodazole or 100 nM taxol for 24 h. After treatment, mitochondria were isolated using a mitochondria isolation kit from Sigma. Whole cell lysates (WC), cytosolic (C) and mitochondrial (M) fractions were then resolved on SDS-PAGE and subjected to western blot analysis using anti p53, β-actin and COX IV antibodies. (**c**) The band intensities from *“a”* were quantified using Image J software and normalized with β-actin (WC and C) or COXIV (M) levels. (Full-length uncropped blots are included in Supplementary Fig. S6) (**d**) H460 cells were either left untreated or treated with 1 uM FZ for 24 h and then processed for JC-1 staining *(i, control; ii, FZ)*.

We earlier reported decreased mitochondrial membrane potential, release of cytochrome c and PARP cleavage in cells undergoing apoptosis in response to FZ treatment. It was shown that FZ treatment resulted in accumulation of p53 protein mainly due to inhibition of proteasomal degradation, and an accumulation of the ubiquitylated form of p53 was also observed^35^. We, therefore, looked at the mitochondrial levels of p53 when NSCLC cells were exposed to the drug. For this p53 null H1299 cells were transfected with GFP-p53 construct and its subcellular distribution was examined following treatment with different microtubule targeting agents for 24 h (Fig. 7a). Western blot for p53 showed an increased level of p53 protein in the mitochondrial fraction following FZ treatment (Fig. 7b,c). Additionally, the p53 translocated to mitochondria in response to FZ treatment appeared to be monoubiquitinated, indicating that the increased pool of monoubiquitinated p53 in FZ treated cells is translocated to mitochondria resulting in the mitochondrial cell death pathway in these cells^40^ (Fig. S3). Correspondingly, mitochondrial membrane depolarization was also observed in FZ treated cells using JC1 voltage sensitive dye (Fig. 7d).

### Inhibition of glucose uptake by FZ sensitizes cancer cells to undergo apoptosis

**Figure 8:**
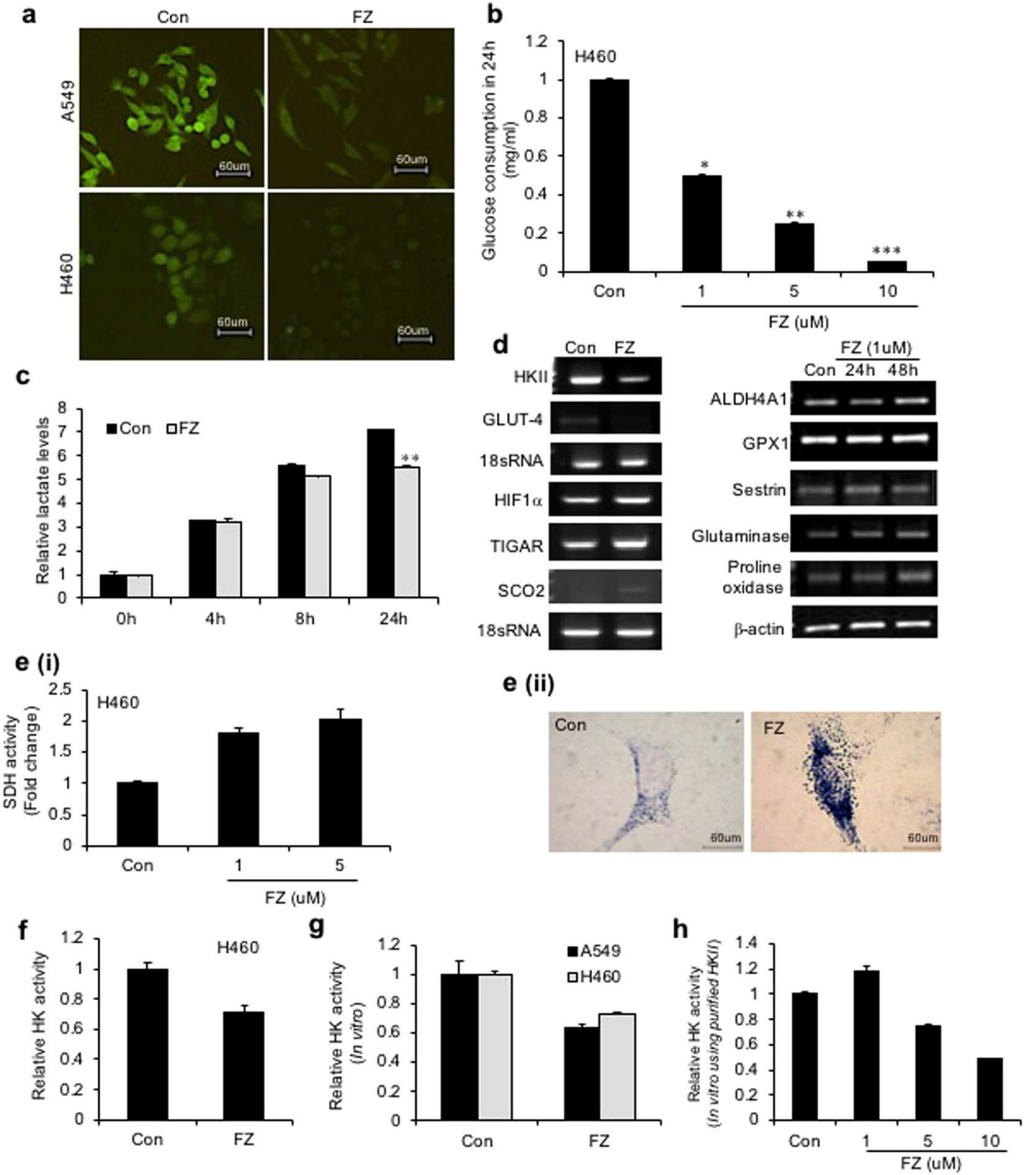
FZ alters glucose uptake and impairs enzymatic activity of HKII in NSCLC cells. (**a**) A549 or H460 cells were treated with 1 uM FZ for 4 h and uptake of the fluorescent glucose derivative 2-NBDG was examined thereafter by fluorescence microscopy as described. Representative images of cells from three independent experiments are shown. (**b**) Human NSCLC H460 cells were exposed to increasing doses of FZ for 24 h. Culture supernatants were then used to assess glucose consumption by glucose oxidation assay using GO assay kit from Sigma. (**c**) H460 cells were left untreated or treated with 1 uM FZ and the lactate levels in culture supernatants were assessed after the indicated time points using Lactate Assay Kit from BioVision. (**d**) Human H460 cells were exposed to 1 uM FZ for 24 or 48 h as indicated, total RNA was isolated and RT-PCR was performed using primers specific for the indicated genes. (**e**) (i) H460 cells were left untreated or treated with 1 uM FZ for 24 h and the cell extracts were then processed for a spectophotometric assay for SDH (A_630nm_). (ii) A549 cells were left untreated or treated with 1 uM FZ for 20 h following which they were processed for histochemical assay to assess SDH activity. Cells were then observed under a microscope and images were acquired at 40X magnification. (**f**) H460 cells were left untreated or treated with 1 uM FZ for 24 h. HK enzymatic activity was then determined spectrophotometrically as described under “Materials and Methods”. (**g**) H460 and A549 cell lysates were incubated with DMSO or FZ for 15 min prior to initiation of reaction. HK enzymatic activity was then determined spectrophotometrically as described under “Materials and Methods”. (**h**) Purified HKII from *S. cerevisae* was incubated with increasing doses of FZ and HK activity was then measured spectrophotometrically. (*p < 0.05, **p < 0.01, ***p < 0.005).

In studies with parasites, the anthelmintic effects of the benzimidazoles have been related to inhibition of glucose uptake with resultant alterations in glucose metabolism^41^. We tested the effect of FZ on glucose uptake in human cancer cells. H460 and A549 cells were treated with 1 uM FZ for 4 h and uptake of fluorescent glucose analogue 2-NBDG was observed. FZ treatment resulted in inhibition in glucose uptake in both the cell lines (Fig. 8a). Similar results were obtained when a glucose oxidation assay was performed using culture supernatants from cells treated with increasing concentrations of FZ (Fig. 8b). Expectedly, FZ treatment also resulted in reduced lactate levels (Fig. 8c). Hence, FZ induced cell death appeared to be related to inhibition of glucose uptake.

Since an increase in soluble tubulin, and both p53 induction and NFκB downregulation [reported earlier^35^], have been linked with glucose metabolism, the expression of genes involved in the process was examined. An increase was observed in the mRNA levels of *Glutaminase 2* and *Proline oxidase* 48 h after FZ treatment while *GLUT-4* and *Hexokinase II* showed considerably reduced expression. p53 inducible pro-apoptotic genes, *TIGAR* and *SCO2* were also induced to an appreciable extent following FZ treatment (Figs 8d and S4).

Hexokinase II (HKII), a key glycolytic enzyme, plays a critical role in glucose retention and metabolism and is highly advantageous for cancer cell survival and proliferation. Therefore, we examined whether FZ impairs the enzymatic function of HKII in cancer cells. An enzymatic assay for HKII activity was performed after treating NSCLC cells with FZ for 24 h (Fig. 8f). The results showed reduced HKII activity in FZ treated cells, suggesting the inhibition of HKII activity to be a causative factor for reduced glucose uptake in FZ treated cells, eventually leading to the activation of apoptotic signals. *In-silico* models indicated that this action may be due to the ability of FZ to mimic glucose or glucose-6-phosphate (G6P) by stably binding to its pocket in HKII (Fig. S5 and Table S1). The result was further validated by *in vitro* hexokinase assays (Fig. 8g and h).

Various benzimidazole compounds have been shown to be highly effective as inhibitors (up to 50% reduction of activity) of the helminth-specific enzyme fumarate reductase *in vitro*. While fumarate reductase converts fumarate to succinate in microbes and lower organisms, succinate dehydrogenase catalyzes the oxidation of succinate to fumarate in mammalian mitochondria. The two enzymes, therefore, act on the reverse directions of the same enzymatic interconversion. Succinate dehydrogenase (SDH) is a mitochondrial tricarboxylic acid (TCA) cycle enzyme and a known tumour suppressor gene^42^. Succinate, a TCA cycle metabolite, is accumulated due to *SDH* downregulation and provides cancer cells with a growth advantage leading to tumour progression^42^. Spectrophotometric assay as well as histochemical staining^43^ showed enhanced SDH activity in cells following FZ treatment (Fig. 8e *i* and *ii*).

### Combination effect of FZ with other drugs

Finally, we evaluated the effect of FZ in combination with the microtubule targeting drug taxol, glycolytic inhibitor 2 deoxyglucose (2DG) and dichloroacetate (DCA) - a pyruvate dehydrogenase kinase inhibitor which acts by shifting the metabolism towards glucose oxidation over glycolysis. In order to determine whether FZ could show an additive or synergistic effect with these drugs, cell proliferation assays were performed and the combination effects were analyzed using the combination index (CI) method^44^. The Fa-CI plot for the combinations showed that the CI values were <1 over the entire range for combination with DCA, CI value was 0.04 at the 50% effective dose, suggesting a strong synergism by FZ and DCA. (Fig. S2-a) Similarly, CI values for the FZ-2DG combination were also less than 1 for the higher doses with a CI of 0.21 at the Fa value 0.5. (Fig. S2-b). CI <1 was also observed at most doses of FZ-Taxol combination (CI-0.52 at 50% inhibition). (Fig. S2-c). Since CI <1 represents synergism as per the CI method, we conclude that FZ shows synergistic effect with DCA, 2DG and over a range of doses with taxol.

### FZ effectively inhibits colony formation of human NSCLC cells in culture

**Figure 9:**
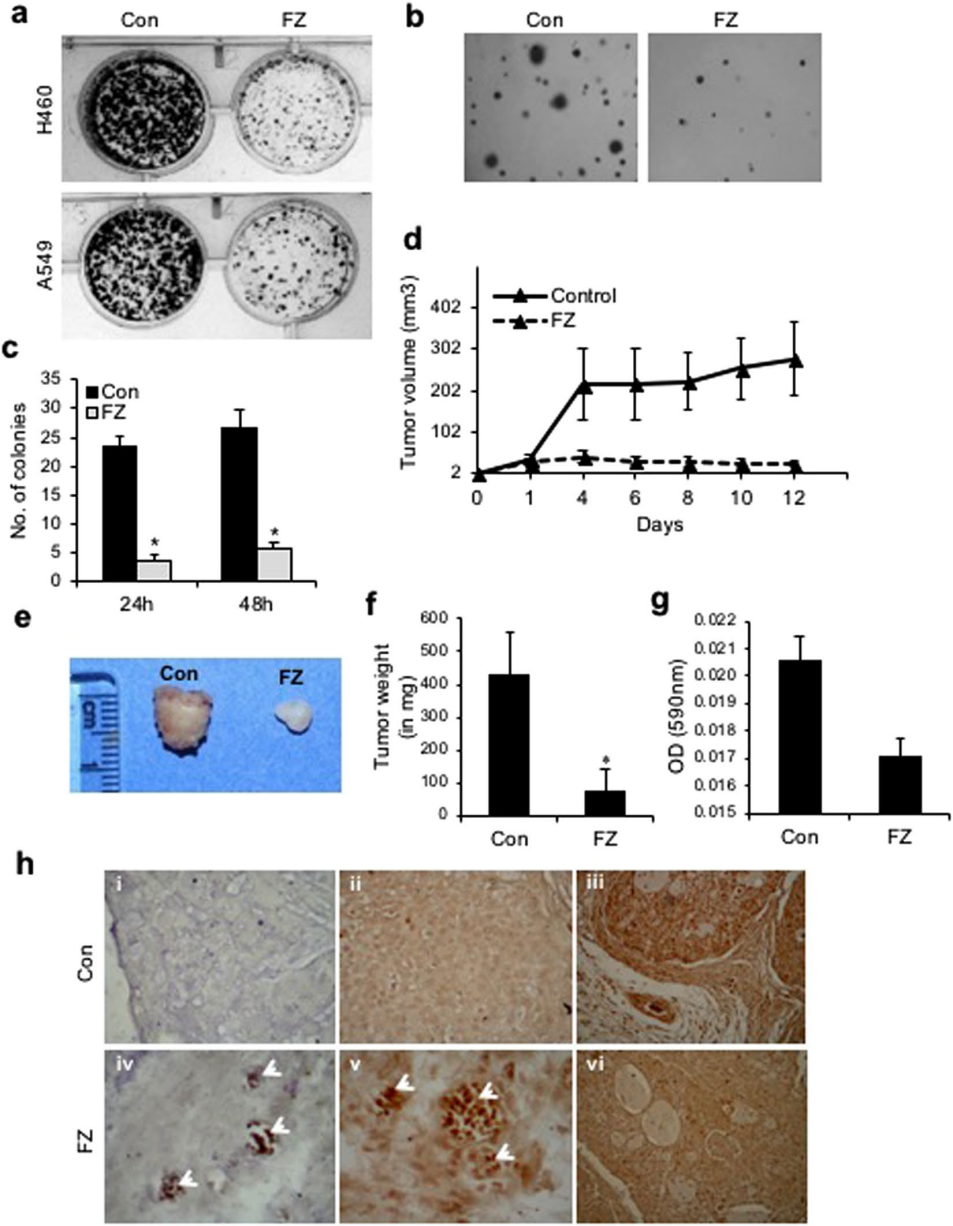
FZ treatment results in reduced tumorigenicity *in vitro* and *in vivo*. (**a**) Colony formation assay for H460 and A549 cells following treatment with 1 uM FZ for 48 h. (**b**) Soft agar assay following FZ treatment. Cell colonies were counted after staining with 5% crystal violet (**c**). (**d**) Tumors were established in *nu/nu* mice by subcutaneous injection of 5 × 10^6^ A549 cells. After the tumors were 2–4 mm in diameter, the mice were orally fed with FZ dissolved in olive oil (1 mg/mouse) every second day, whereas control animals received olive oil only. Tumor volumes were then calculated by measuring tumor dimensions using Vernier callipers. (**e** and **f**) Tumors were excised, photographed and weighed. (**g**) Tumor vascularity *in vivo* was quantified between control and FZ treated mice by measuring hemoglobin spectrophotometrically (A_590nm_). (**h**) Tumor sections from control untreated and FZ treated mice were processed for TdT staining to identify apoptotic cells (*i & iv*), sections were immunostained using p53 specific antibody (*ii & v*), and immunohistochemistry was performed on sections for CD31 (*iii & vi*). *i, ii & iii* are sections from control mock treated mice and *iv, v & vi* are sections from FZ treated mice (*p < 0.05).

The effect of FZ on cancer cells *in vitro* was examined by the colony forming ability of A549 and H460 cells in culture. Treatment of these cells with 1 uM FZ for 48 h resulted in significant reduction in number of colonies as compared to control untreated cells (Fig. 9a). Further, anchorage independent growth of control and FZ treated H460 cells was evaluated by soft agar assay. Results of the soft agar assay correlated with colony formation data (Fig. 9b,c) suggesting that FZ is a potential antineoplastic agent that kills cancer cells *in vitro*.

### *In vivo* tumour suppression by FZ treatment

The therapeutic activity of FZ *in vivo* was examined by giving oral doses to nude mice bearing A549 xenografts. Female athymic *nu*/*nu* mice were xenografted with A549 cells and mice bearing tumours (2–3 mm) were fed with FZ (1 mg/mouse) orally every second day for 12 days (Fig. 9d). At the end of 12 days, tumours were excised, measured and weighed. FZ administration led to a marked reduction in tumour size and weight (Fig. 9e,f). Further, the tumor vascularity was quantified in control and FZ treated mice by spectrophotometrically measuring hemoglobin content (A_590nm_). FZ administration led to a reduction in hemoglobin content in tumors signifying reduced tumor vascularity (Fig. 9g). TUNEL staining of tumour sections showed a significant number of apoptotic cells (Fig. 9h *i* & *iv*). These results suggest that FZ inhibits tumour cell growth *in vivo* by inducing apoptosis of tumour cells. When tumour sections were further examined for p53 protein expression, a number of p53 positive tumour cells were visible in FZ treated mice suggesting p53 induced cell death (Fig. 9h *ii* & *v*). Moreover, FZ treated A549 tumours showed very few CD31 positive endothelial cells in xenografts (Fig. 9h *iii* & *vi*). These data are in good agreement with our *in vitro* analysis of FZ mediated cell death.

## Discussion

Microtubules are major components of the cytoskeleton and play important roles in a variety of cellular processes like intracellular trafficking, maintenance of cell shape and structure, polarity, cell signalling, and mitosis. Microtubule targeting agents (MTAs) are being used clinically for the treatment of multiple tumour types, but their effectiveness is often largely affected by drug resistance mechanisms. Although the principal mechanism of MTAs’ cytotoxicity relates to their interactions with tubulin and disruption of microtubule function, differences in their tubulin binding characteristics alter their modes of action with a significant impact on the efficacy or toxicity profile of each agent. Newer agents with improved efficacy, tolerability, and the ability to even partially overcome resistance could be of great significance.

The present data reveals FZ as a moderate microtubule targeting agent causing mitotic arrest followed by cancer cell death. Despite being a relatively mild microtubule targeting agent, FZ possesses a unique ability to induce p53 to a considerably high level.

In our earlier work we have shown the activity of FZ as a proteasomal interfering agent^35^. The present study revealed an early elevation and stabilization of cyclin B1 levels in response to FZ, indicating a progression of cells towards cell death rather than mitotic exit and polyploidy. Interestingly, the FDA approved proteasomal inhibitor bortezomib has also been reported to induce mitotic cell death in lymphoma cells^45^. Therefore, proteasomal inhibition associated with mitosis-selective therapeutic approach adds to the significance of FZ as a potential anti-cancer agent.

Benzimidazole compounds are known to interfere with energy metabolism of the host, particularly, the carbohydrate metabolism. They block the glucose uptake and ATP formation which ultimately leads to the death of the parasite. Disparity in energy metabolism between normal and cancer cells has been well known for a long time and in many malignant cell lines glucose consumption is several folds higher than in normal cells. Deprivation of glucose uptake, therefore, has been exploited as a therapeutic approach^46^. FZ could effectively inhibit glucose uptake in NSCLC cells suggesting that FZ induced cancer cell death is, in part, facilitated by blocking glucose uptake of cancer cells.

Enhancement of oxidative phosphorylation and/or inhibition of glycolysis by certain agents (e.g., dichloroacetic acid, 3-bromopyruvate) have been reported to result in tumor cell death^47,48^. Since unlike normal cells, cancer cells mostly thrive on increased glycolysis for generation of ATP, impairment of this pathway could lead to fatal consequences specifically for these cells. Also, it has been postulated that free tubulin regulates mitochondrial function in cancer cells but not in non-transformed primary cells, via alteration of mitochondrial membrane potential^49^. Therefore, microtubule targeting agents having the ability to affect glucose metabolic pathways can be immensely beneficial as anti-cancer agents. FZ exposure reduced the expression of *Glut-4* transporter as well as *hexokinase (HK II)*, which may be linked to p53 activation and alteration of microtubule dynamics^50,51^. Hexokinase II is a key glycolytic enzyme, which, besides acting to promote glycolysis in co-operation with the GLUT transporters, also acts to suppress mitochondria-induced apoptosis^52^. Targeting this crucial enzyme is therefore being investigated as a possible strategy to effectively curb cancer cell growth. Similarly, *proline oxidase*^53,54^, *SCO2*^55,56^, *TIGAR*^42,57,58^ and *glutaminase 2* (GLS2)^59^ are all p53 target genes involved in the regulation of cellular metabolism which were found to be induced following FZ treatment. Therefore, tubulin depolymerization and p53 induction caused by FZ may further be leading to modulation of glucose uptake as well as glycolytic pathway.

Altogether, our findings show microtubule disruption, p53 stabilization and interference with glucose metabolism as collective underlying mechanisms of FZ induced preferential elimination of cancer cells both *in vitro* and *in vivo*.

## Materials

Fenbendazole (FZ), 3-(4,5-dimethylthiazol-2-yl)-2,5-diphenyltetra- zolium bromide (MTT), colchicine, taxol, JC-1 (5,5′,6,6′-tetrachloro-1,1′,3,3′-tetraethyl-benzimidazoleocarbocyanine iodide), Hoechst 33342, propidium iodide, anti β-actin, anti-mouse IgG-fluorescein isothiocyanate (FITC), horseradish peroxidase (HRP) conjugated anti-mouse, anti-rabbit and anti-goat IgGs, TRI reagent, purified HKII from *S. cerevisae* as well as all the cell culture reagents were purchased from Sigma. Nocodazole was obtained from Calbiochem. 2-(*N*-(7-nitrobenz-2-oxa-1,3-diazol-4-yl)amino)-2-deoxyglucose (2-NBDG) and MitoTracker were purchased from Molecular Probes. Anti p53 (Bp53-12 and DO1), anti-p21, anti MDM-2, anti α-tubulin, anti Ac-α-tubulin, anti pH3 (Ser10) and anti-cyclin B1 antibodies were purchased from SantaCruz Biotechnology.

### Cell lines

All cell lines were procured from NCCS, Pune except H1299 which was kindly provided by Dr. Bert Vogelstein. The cells were grown in Dulbecco’s modified Eagle’s medium (DMEM) supplemented with 10% heat-inactivated fetal bovine serum (FBS) and 1X penicillin/streptomycin antibiotics (100 u/ml penicillin and 100 ug/ml streptomycin).

## Methods

### Immunofluorescence for tubulin

Tubulin organization following FZ treatment was visualized by immunofluorescence using anti α-tubulin antibody. Human NSCLC A549 cells were grown on coverslips and treated with 1 uM FZ or 0.05 ug/ml colchicine for 24 h. Following treatment, cells were rinsed twice with PEM-PEG buffer (80 mM PIPES, 1 mM EGTA, 0.5 mM MgCl_2_, 4% PEG-8000) and permeabilized with PEM-PEG buffer containing 0.05% Triton X-100. The cells were then rinsed with PEM-PEG buffer and quickly fixed in 3% formaldehyde in PEM with 1% DMSO for 30 min at RT. After washing with PEM-PEG buffer, primary antibody (anti α-tubulin) incubation was carried out overnight at 4 °C. After washing with PBS, cells were incubated with FITC-conjugated secondary antibody for 2 h at 37 °C, washed several times, and visualized using a fluorescence microscope. Nuclei were counter-stained red using propidium iodide.

### Fractionation of soluble and polymerized tubulin

Separation of soluble and polymerized tubulin fractions from A549 cells was carried out as described by Legault *et al*.^60^. After drug exposure, about 5 × 10^6^ cells in 100 mm petridishes were washed with PBS at 37 °C and harvested in 1 ml of PBS containing 0.4 ug/ml of paclitaxel using a rubber policeman. Cells were then centrifuged and lysed using 250 ul of microtubule stabilizing buffer [20 mM Tris-HCl (pH 6.8), 140 mM NaCl, 1 mM MgCl_2_, 2 mM EDTA, 0.5% NP40 and 0.4 ug/ml paclitaxel] and centrifuged at 12,000 × g for 10 min at 4 °C. The supernatants containing soluble tubulin were mixed with 2X Laemmli’s sample buffer. Pellets containing the polymerized tubulin were resuspended in 250 ul of water, followed by two freeze/thawing cycles and finally resuspended in Laemmli sample buffer. Samples were analyzed by western blot using anti α-tubulin antibody.

### Tubulin polymerization assay

Polymerization of bovine tubulin was measured according to Beyer *et al*.^61^. Briefly, bovine tubulin (1.8 mg/mL; Sigma) was added to ice-cold polymerization buffer (PEM: 80 mM PIPES, 0.5 mM EGTA, 2 mM MgCl_2_, 10% glycerol, and 1 mM GTP) and centrifuged at top speed in a microcentrifuge for 5 minutes at 4 °C. Supernatant (100 μL/well) was immediately added to a 96-well plate, which contained 10 uM FZ or dimethyl sulfoxide control in PEM buffer. After addition of tubulin, the plate was immediately placed in the spectrophotometer (Tecan multimode reader), which was maintained at 37 °C, and the absorbance was measured every 5 minutes for 2.5 hours at 340 nm.

### Competitive tubulin binding assay

10 uM FZ was coincubated with 3 μM colchicine in PEM buffer containing 3 μM tubulin at 37 °C for 60 min. After incubation, the fluorescence of tubulin-colchicine complex was measured using a Tecan multimode reader at excitation wavelength of 380 nm and emission wavelength of 435 nm. PEM buffer was used as a blank. The raw fluorescence values were normalized by setting the fluorescence of 3 μM tubulin with 3 μM colchicine to 100%.

### Rhodamine 123 accumulation assay

Cells were seeded in a 6 well plate and incubated with 0.5 uM FZ for 6 or 24 h with or without 10 μM verapamil. Following day, Rho 123 (10 μM) was added and the cells were further incubated for 1 h at 37 °C in the dark. Cells were then washed thoroughly three times with ice cold PBS and images were acquired using a fluorescence microscope. Alternatively, fluorescence was measured at Ex507/Em529 on a Tecan Infinite M200 multimode plate reader.

### FACS analysis

Cells were synchronized by serum starvation for 48 h. After 48 h, the media were replaced with fresh media containing serum and cells were treated with 1 uM FZ for the indicated time intervals. Following treatment, cells were harvested, washed with PBS and fixed in 70% ethanol overnight at 4 °C. Next day, cells were centrifuged at 1000 rpm for 5 min, the supernatant was carefully aspirated and the pellet was resuspended in PBS. The cells were again centrifuged, the supernatant removed and the pellet was finally resuspended in PBS containing 40 ug/ml PI and 100 ug/ml RNase A. FACS analysis was done on a BD FACS Array.

### RT-PCR and qPCR

Cells were treated with FZ as indicated and total RNA was extracted using TRI reagent from Sigma. Concentrations of RNA in different samples were determined using a spectrophotometer. RNA was reverse transcribed using oligo (dT)_18_ primers and RT-PCR analysis was performed. For qPCR, RealMasterMix SYBR ROX kit from Eppendorf was used. The reactions were set up according to the manufacturer’s instructions in an Eppendorf Mastercycler Realplex real-time PCR machine.

### Immunoblotting experiments

After treatment as indicated, the total cell lysates or the sub-cellular fractions were separated through 10% SDS-polyacrylamide gel electrophoresis and transferred onto polyvinylidene difluoride (PVDF) membranes. Protein concentration was measured according to the method of Bradford using bovine serum albumin as a standard^62^. The membranes were successively incubated in blocking buffer [5% skimmed milk in TBST (50 mM Tris, pH 7.5, 0.15 M NaCl, 0.05% Tween-20)], with primary antibody and then with secondary antibody conjugated with horseradish peroxidase. Detection was carried out using enhanced chemiluminiscense reagent from Millipore. All primary antibodies were used in 1:4000 dilutions for immunoblotting.

### Glucose uptake assay

Glucose uptake assay was performed using 2-[N-(7-nitrobenz-2-oxa-1,3-diazol-4-yl)amino]−2-deoxy-D-glucose (2-NBDG; Invitrogen), a fluorescent analogue of 2- deoxyglucose. After overnight culture, the cells were left untreated or treated with 1 uM FZ for 4 h following which they were pre-incubated in glucose free KRB buffer (129 mM NaCl, 5 mM NaHCO_3_, 4.8 mM KCl, 1.2 mM KH_2_PO_4_, 1.0 mM CaCl_2_, 1.2 mM MgSO_4_, 10 mM HEPES, 0.1% BSA) pH 7.4 for 15 min at 37 °C. The cells were then incubated in fresh KRB buffer supplemented with 400 μM 2-NBDG and 3.3 mM glucose for 10 min at 37 °C. Cells were observed and imaged under a fluorescence microscope after washing with KRB buffer.

### Glucose oxidation assay

Glucose utilization was estimated in cells exposed to FZ using the commercially available glucose assay kit from Sigma as per manufacturer’s instructions. After 24 h of drug exposure, culture supernatants were collected and centrifuged to remove any cellular debris. Assay reagent was mixed with the culture supernatants and incubated at 25 °C for 30 min, after which the absorbance was recorded at 505 nm on a PerkinElmer VictorX3 spectrophotometer.

### Assays for succinate dehydrogenase activity

For spectophotometric determination of SDH activity, 20ul buffer B (200 mM Na-phosphate buffer, pH 7.4),10 ul 2.5 mg/ml NBT, 10 ul 1% Triton-X 100 and 10 ul substrate B (100 mM Na succinate, pH 7.4) were added to the enzyme fraction (cell lysates). The mixture was incubated at 37 °C for 30 min and the reaction was stopped by adding 40ul of 10% SDS. Absorbance was then measured at 630 nm.

For histochemical staining, A549 cells were plated onto coverslips and cultured overnight. After treatment, cells were washed with 1 mM malonate in 0.9% NaCl. The cells were then fixed in acetone for 5 min at −20 °C. After fixing, they were coated with CoQ_10_ (0.2 mg/ml in acetone) and incubated for 1 h at 37 °C in 50 mM succinate and 0.5 mg/ml NBT in 200 mM phosphate buffer (pH 7.6). The staining was observed and images acquired under a bright field microscope.

### Enzymatic assay for hexokinase

Enzymatic assay for determination of hexokinase activity of control and FZ treated cells was performed as described by Darrow and Colowick^63^ by using glucose as substrate. The final concentrations of the reaction mixture were 8.3 mM glycylglycine, 17 mM ATP, 0.0011% cresol red, 14 mM magnesium chloride, 27 mM glucose. For determining HK activity, the reaction mix was added to the cellular extracts and the decrease in A_560nm_ was measured for approximately 5 minutes.

### Colony formation assays

H460 and A549 cells were seeded in a 12-well plate in triplicate (500 cells/well) and left untreated or treated with 1 uM FZ for 48 h. After 48 h, the cells were washed with PBS and the media was replaced with fresh media. The cells were allowed to grow for 8 days in order to form colonies and then stained with Coommassie Brilliant Blue. Anchorage-independent growth was assayed by the ability of cells to grow in soft agar. 5 × 10^3^ untreated or FZ treated A549 cells (1 uM, 48 h) were overlayed in a 0.3% agarose solution in DMEM on a layer of 0.6% agarose in a 6-well plate and incubated for 14 d to allow colony formation. Cell colonies were then counted after staining with 5% crystal violet.

### Nude mice experiments

Human NSCLC A549 cells were grown in DMEM with 10% FBS. When cells were 70–80% confluent, 3–4 h before harvesting, medium was replaced with fresh medium to remove dead and detached cells. Cells were then trypsinized and collected in complete medium. They were collected by centrifugation at 1500 rpm for 2–5 min and washed twice with PBS. Cell viability was assessed by trypan blue staining and cell number was determined using a hemocytometer. Cells were resuspended in a volume so that 100 µl contained 5.0 × 10^6^ cells. *nu/nu* mice used were 6 weeks old and they were acclimatization for 3 days before injection. Cells (5.0 × 10^6^) were injected subcutaneously into the right flank of mice. Oral dosing of FZ (1 mg/mouse) was started after 4 weeks when the tumours had reached an average volume of 2–3 mm^3^. Tumour diameters were measured with digital callipers, and the tumour volume in mm^3^ was calculated by the formula: Volume = (width) 2 × length/2.

Experiments on animals in this study were carried out after approval from the Institutional Animal Ethics Committee (IAEC- ACTREC, Mumbai, India). All methods were performed in accordance with the institutional guidelines and regulations.

### Statistical Analysis

All results are expressed as means ± S.D. unless otherwise mentioned. Student’s *t* test was used to calculate the significance, accepting *p* < 0.05 as the level of significance.

## Electronic supplementary material

Fenbendazole acts as a moderate microtubule destabilizing agent and causes cancer cell death by modulating multiple cellular pathways.
