# Supplementary material for: Fenbendazole acts as a moderate microtubule destabilizing agent and causes cancer cell death by modulating multiple cellular pathways
Source: Sci Rep. 2018 Aug 9;8:11926. doi: 10.1038/s41598-018-30158-6 (PMC6085345; doi:10.1038/s41598-018-30158-6)
Supplement: Supplementary file 1 — Fenbendazole acts as a moderate microtubule destabilizing agent and causes cancer cell death by modulating multiple cellular pathways. [file 41598_2018_30158_MOESM1_ESM.pdf]

# Fenbendazole acts as a moderate microtubule destabilizing agent and causes cancer cell death by modulating multiple cellular pathways.

**Nilambra Dogra[1]*, Ashok Kumar[1]#, Tapas Mukhopadhyay[1]**

From the [1]National Centre for Human Genome Studies and Research, Panjab University, Chandigarh-14, India.

## SUPPLEMENTARY INFORMATION

# Figure S1

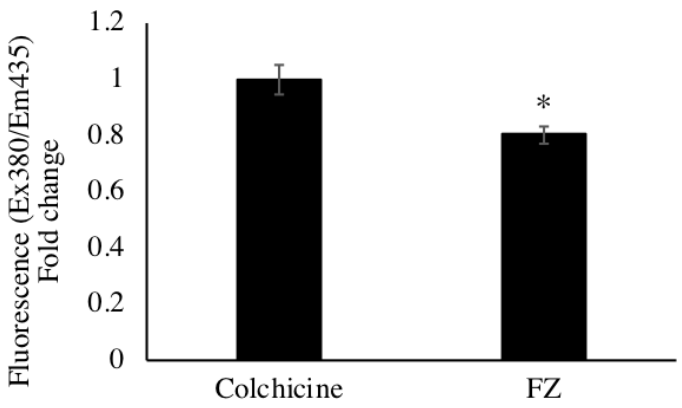


**Fig. S1.** Colchicine binding site assay for FZ.

# Figure S2

**a**

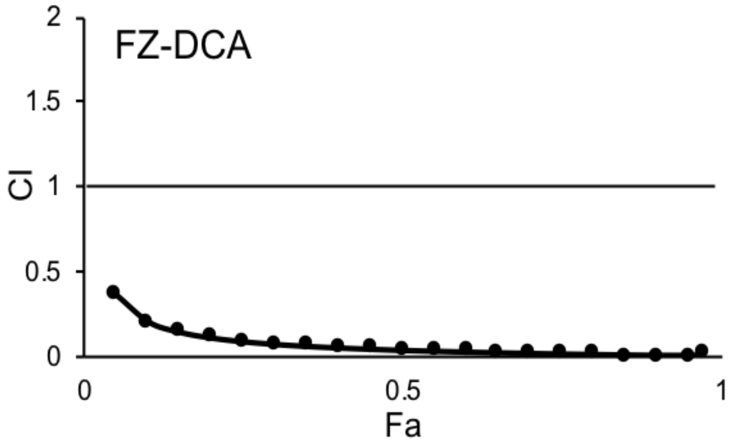


| **Total Dose (mM)** | **Fa** | **CI Value** | **Interpretation** |
|---|---|---|---|
| 0.1001 | 0.09 | 0.42104 | Synergism |
| 0.5005 | 0.25 | 0.12190 | Synergism |
| 1.001 | 0.49 | 0.02043 | Synergism |
| 5.005 | 0.75 | 0.00731 | Synergism |
| 10.01 | 0.74 | 0.01647 | Synergism |
| 50.05 | 0.85 | 0.01788 | Synergism |
| 100.1 | 0.9 | 0.01409 | Synergism |

**b**

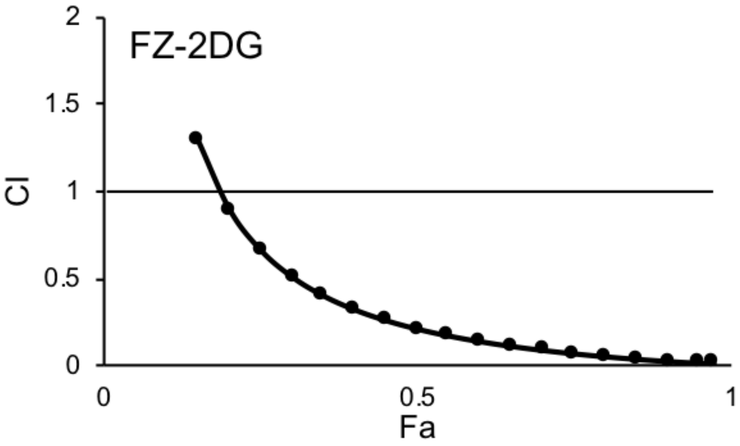


| **Total Dose (mM)** | **Fa** | **CI Value** | **Interpretation** |
|---|---|---|---|
| 0.1001 | 0.011 | 80.8034 | Antagonism |
| 0.5005 | 0.16 | 0.91456 | Additive |
| 1.001 | 0.45 | 0.08042 | Synergism |
| 5.005 | 0.74 | 0.02800 | Synergism |
| 10.01 | 0.72 | 0.06950 | Synergism |
| 25.05 | 0.76 | 0.11168 | Synergism |
| 50.1 | 0.85 | 0.06543 | Synergism |

**c**

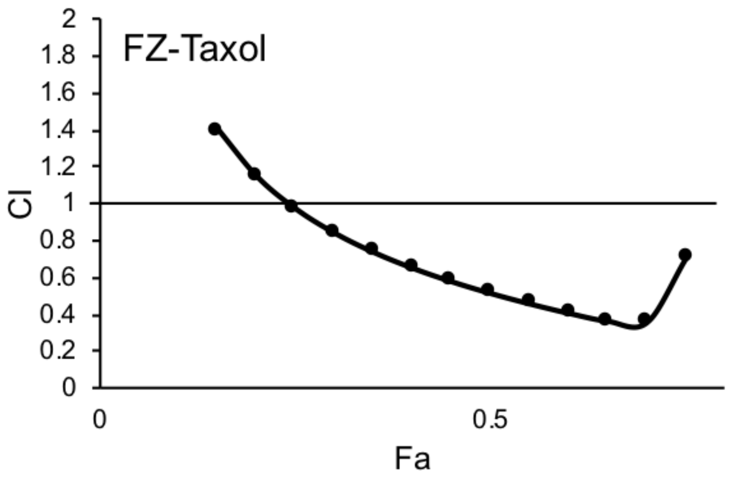


| Total Dose (uM) | Fa | CI Value | Interpretation |
|---|---|---|---|
| 0.11 | 0.63 | 1.05408 | Additive |
| 0.55 | 0.71 | 0.15952 | Synergistic |
| 1.1 | 0.72 | 0.21488 | Synergistic |
| 5.5 | 0.74 | 0.55016 | Synergistic |
| 55.0 | 0.77 | 3.03667 | Antagonistic |
| 110.0 | 0.8 | 4.82115 | Antagonistic |

**Fig. S2.** Combination index (CI) for treatment in A549 cells. The CI was determined using the Compusyn software to evaluate the interaction of FZ with the indicated drugs. A solid horizontal line marks CI = 1. The CI can be interpreted as additive (CI = 1), synergism (CI < 1), and antagonism (C > 1) in drug combinations.

## Figure S3

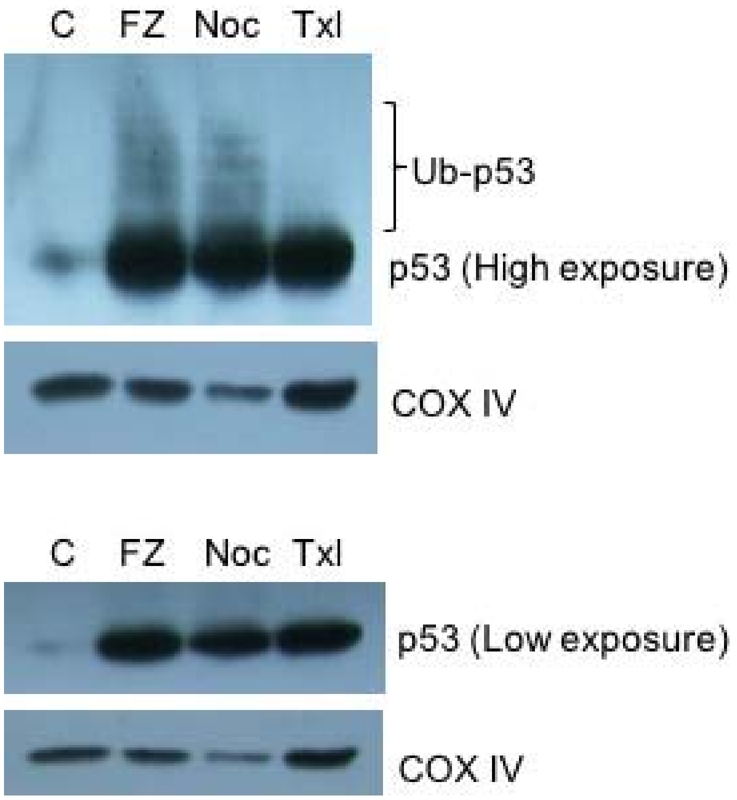


**Fig. S3. Mitochondrial p53 after treatment with different MTAs for 24h.** The mitochondrial fractions from H460 control and treated cells were subject to western blot analysis using anti p53 antibody. The blot was over-exposed for detection of ubiquitinylated bands.

# Figure S4

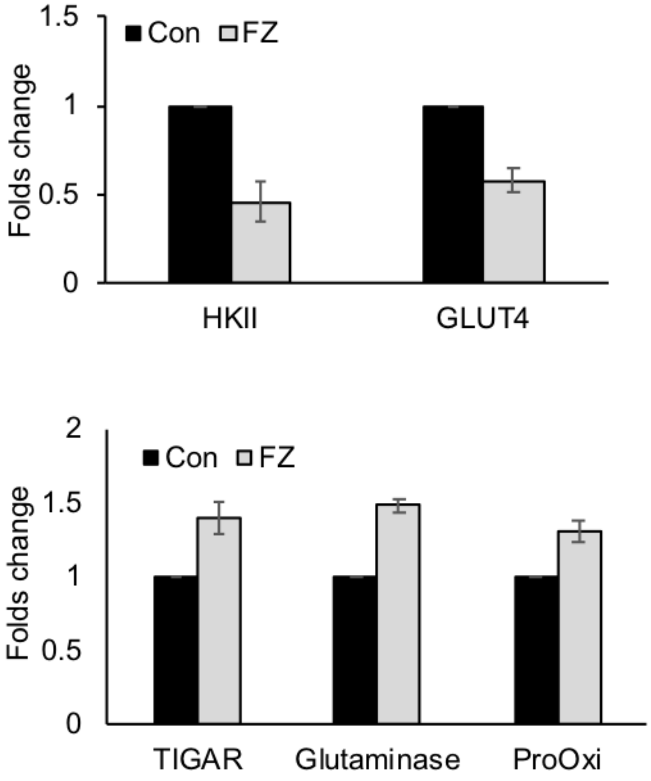


**Fig. S4. Change in expression of p53 modulated metabolic genes following FZ treatment.** Human H460 cells were exposed to 1uM FZ for 24 or 48h as indicated, total RNA was isolated and RT-PCR was performed using primers specific for the indicated genes. Bands obtained in were quantified using ImageJ software.

# Figure S5

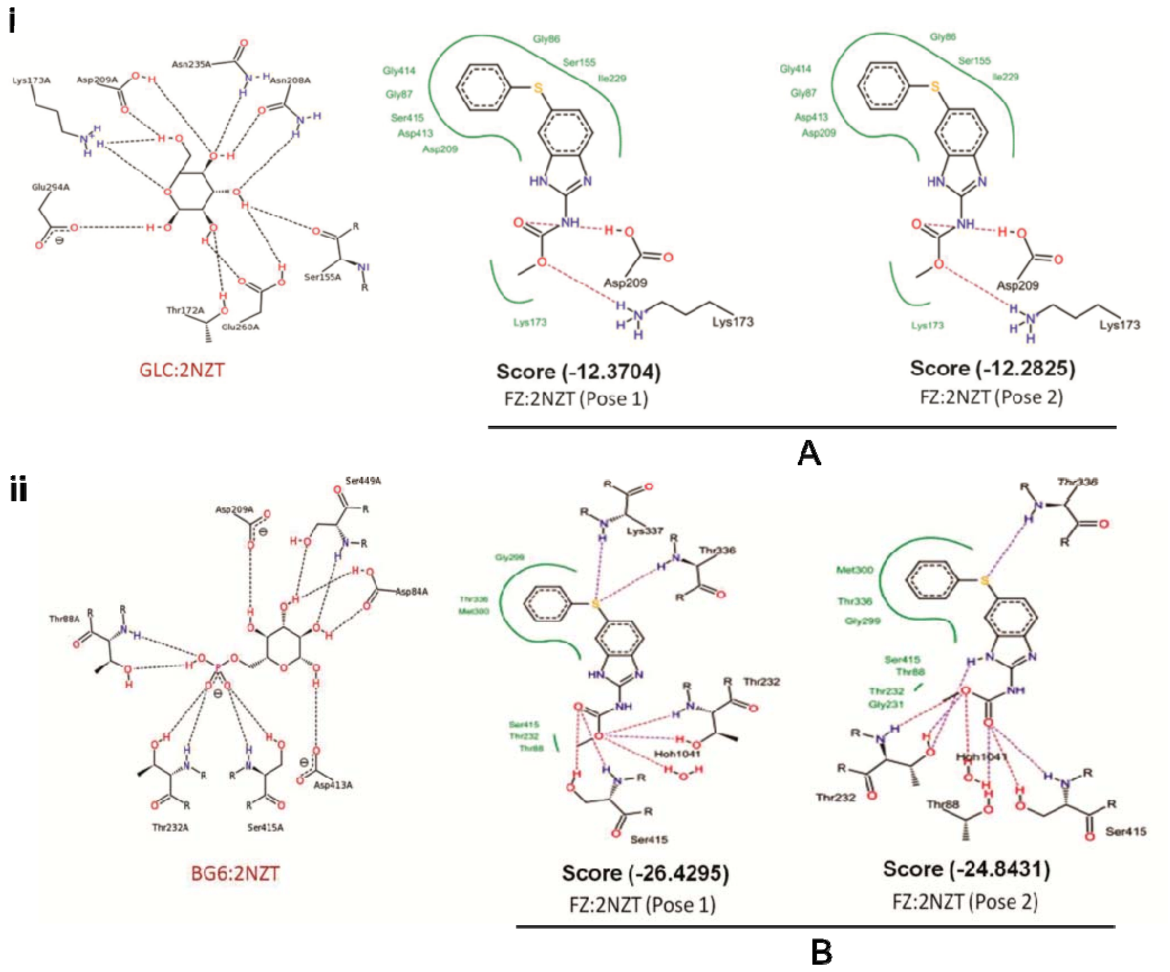


**Fig. S5.** *(i)*, Representative binding modes of FZ at the alpha-D-glucose (GLC) binding site predicted using FlexX software. [2NZT: human hexokinase II from PDB] A, Predicted GLC:2NZT interactions from co-crystallized structure. B, Representative poses showing FZ:2NZT predicted interactions at the GLC binding site. *(ii)*, Representative binding modes of FZ at the beta-D-glucose-6-phosphate (BG6) binding site predicted using FlexX software. [2NZT: human hexokinase II from PDB] A, Predicted BG6:2NZT interactions from co-crystallized structure. B, Representative poses showing FZ:2NZT predicted interactions at the BG6 binding site.

# Table S1

| Ligand | Pose | Score | Hydrogen bonding | Hydrophobic interactions | Interactions of co-crystallized ligand |
|---|---|---|---|---|---|
| **GLC replaced with FZ** | Pose 1 | -12.3704 | **Lys 173**<br>**Asp 209** | Gly 414<br>Gly 87<br>Ser 415<br>Asp 413<br>Asp 209<br>Gly 56<br>**Ser 155**<br>Ile 229<br>**Lys 173** | **Lys173**<br>**Asp209**<br>Asn235<br>Asn208<br>Glu294<br>Thr172<br>Glu260<br>**Ser155** |
| **GLC replaced with FZ** | Pose 2 | -12.2825 | **Lys 173**<br>**Asp 209** | Gly 414<br>Gly 87<br>Asp 413<br>Asp 209<br>Gly 86<br>**Ser 155**<br>Ile 229<br>**Lys 173** | **Lys173**<br>**Asp209**<br>Asn235<br>Asn208<br>Glu294<br>Thr172<br>Glu260<br>**Ser155** |
| **BG6 replaced with FZ** | Pose 1 | -26.4295 | Lys 337<br>Thr 336<br>**Thr 232**<br>Ser 414 | Gly 299<br>Thr 336<br>Met 300<br>**Ser 415**<br>**Thr 232**<br>**Thr 88** | Asp 209<br>Ser 449<br>Asp 84<br>Asp 413<br>**Ser 415**<br>**Thr 232**<br>**Thr 88** |
| **BG6 replaced with FZ** | Pose 2 | -24.8431 | Thr 336<br>**Thr 232**<br>**Thr 88**<br>**Ser 415** | Met 300<br>Thr 336<br>Gly 299<br>**Ser 415**<br>**Thr 88**<br>**Thr 232**<br>Gly 231 | Asp 209<br>Ser 449<br>Asp 84<br>Asp 413<br>**Ser 415**<br>**Thr 232**<br>**Thr 88** |

**Table S1.** Comparative scores and interacting residues of predicted FZ : hexokinase II (2NZT) complex at alpha-D-glucose (GLC) and beta-D-glucose-6-phosphate (BG6) binding sites. Residues highlighted in red are in common with those of the co-crystallized ligand.

## Figure S6

### Fig.1c

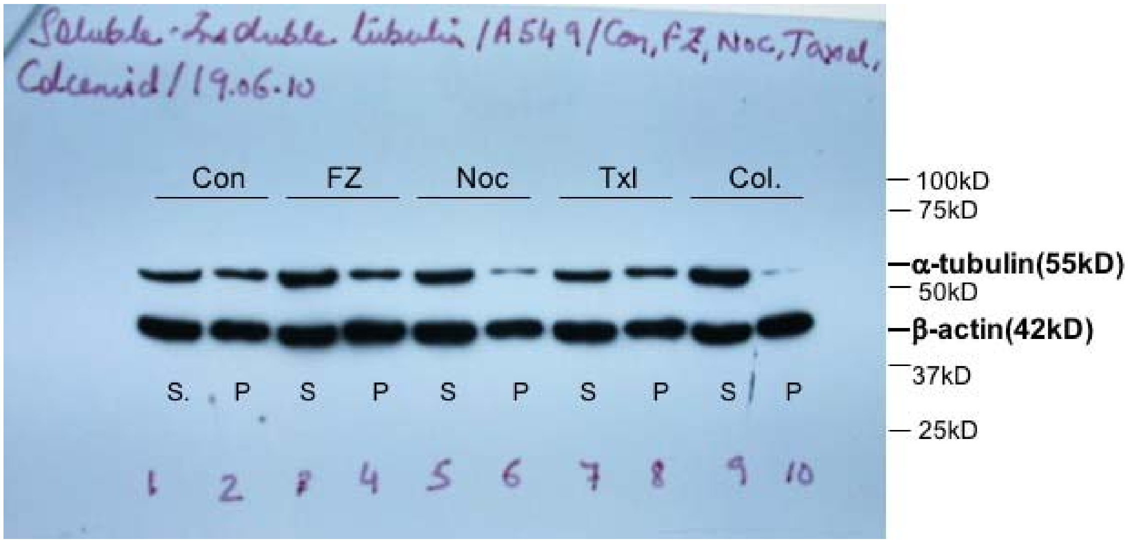


### Fig.1e

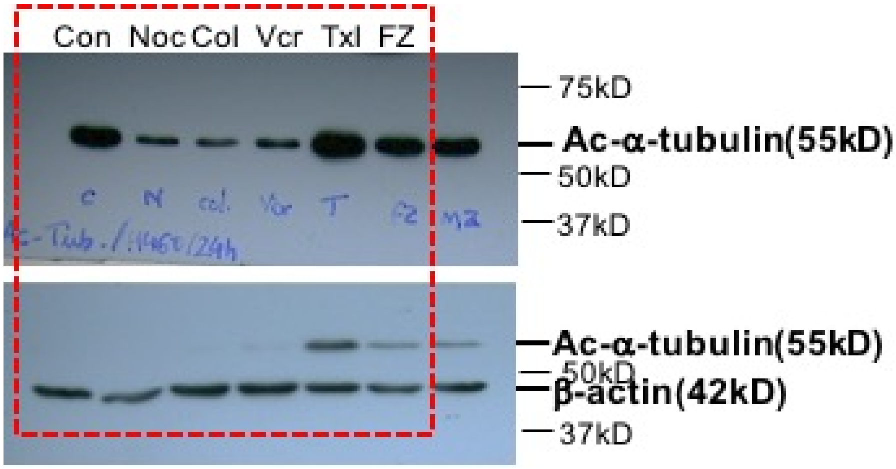

**Fig.3a**

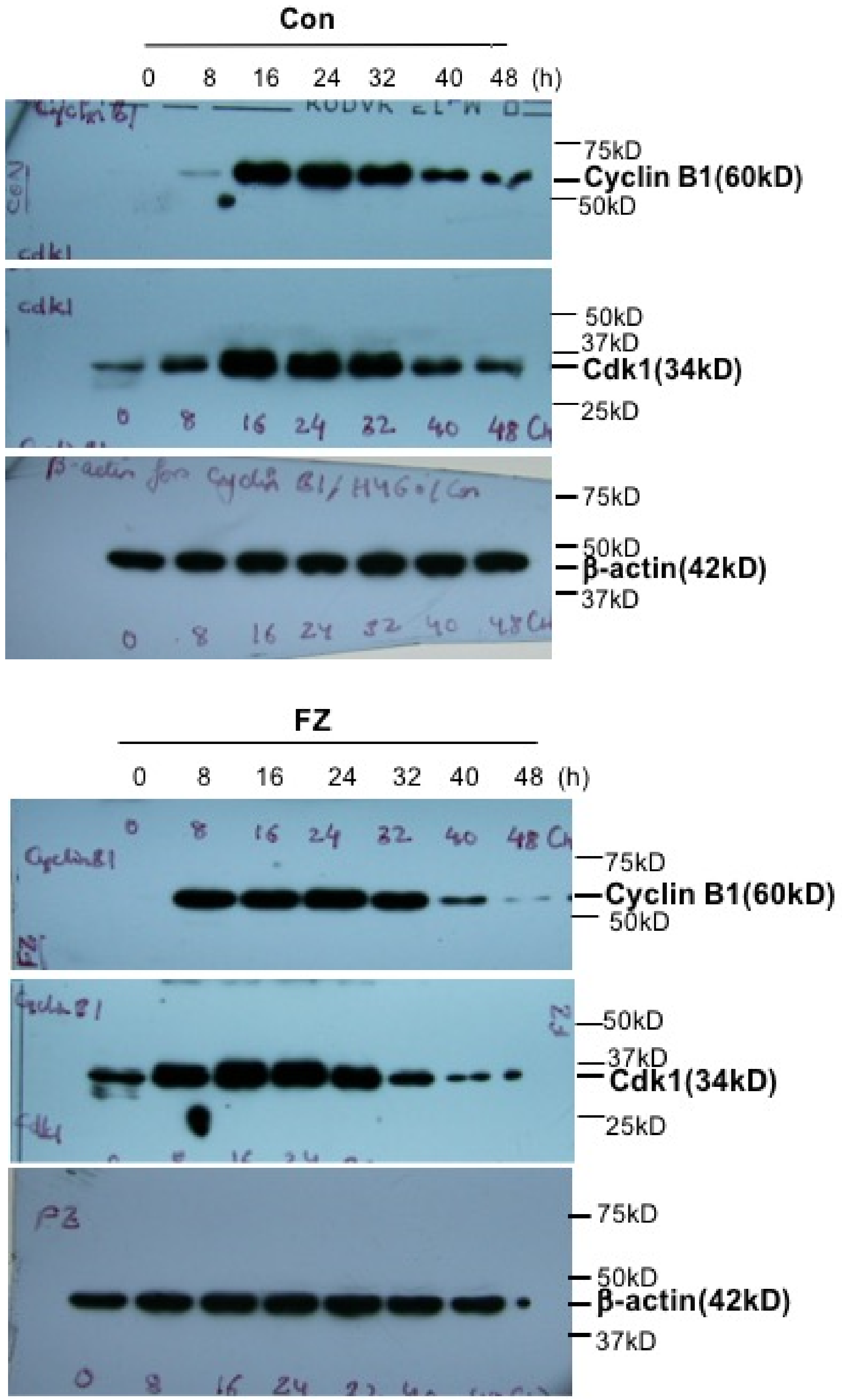
Con
0 8 16 24 32 40 48 (h)
75kD
Cyclin B1(60kD)
50kD
50kD
37kD
Cdk1(34kD)
25kD
75kD
50kD
β-actin(42kD)
37kD
FZ
0 8 16 24 32 40 48 (h)
75kD
Cyclin B1(60kD)
50kD
50kD
37kD
Cdk1(34kD)
25kD
75kD
50kD
β-actin(42kD)
37kD

**Fig.3a contd.**

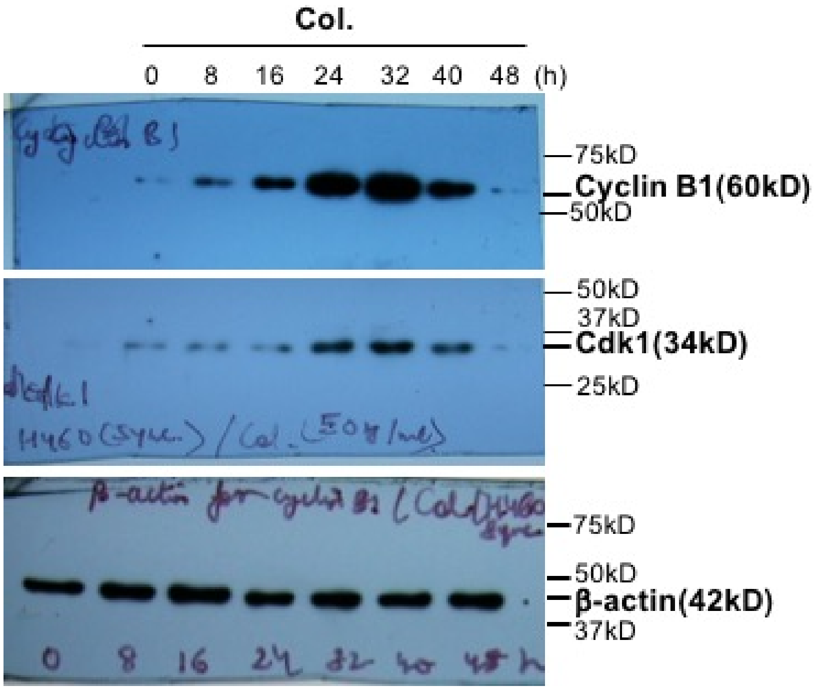


**Fig.3b**

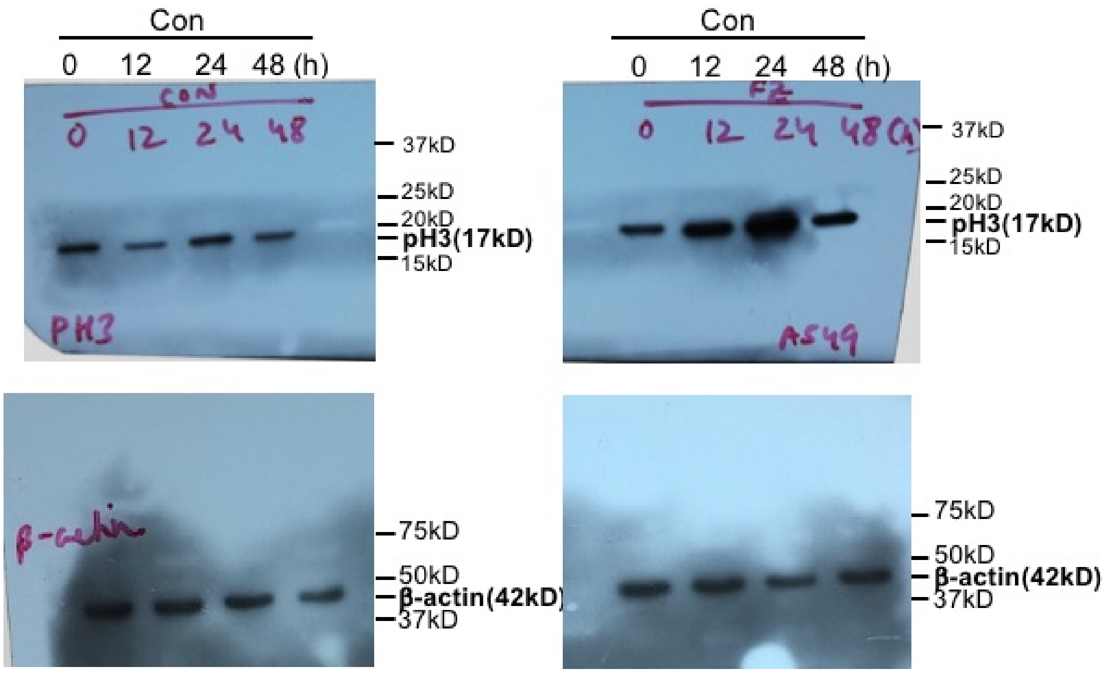

**Fig.6b**

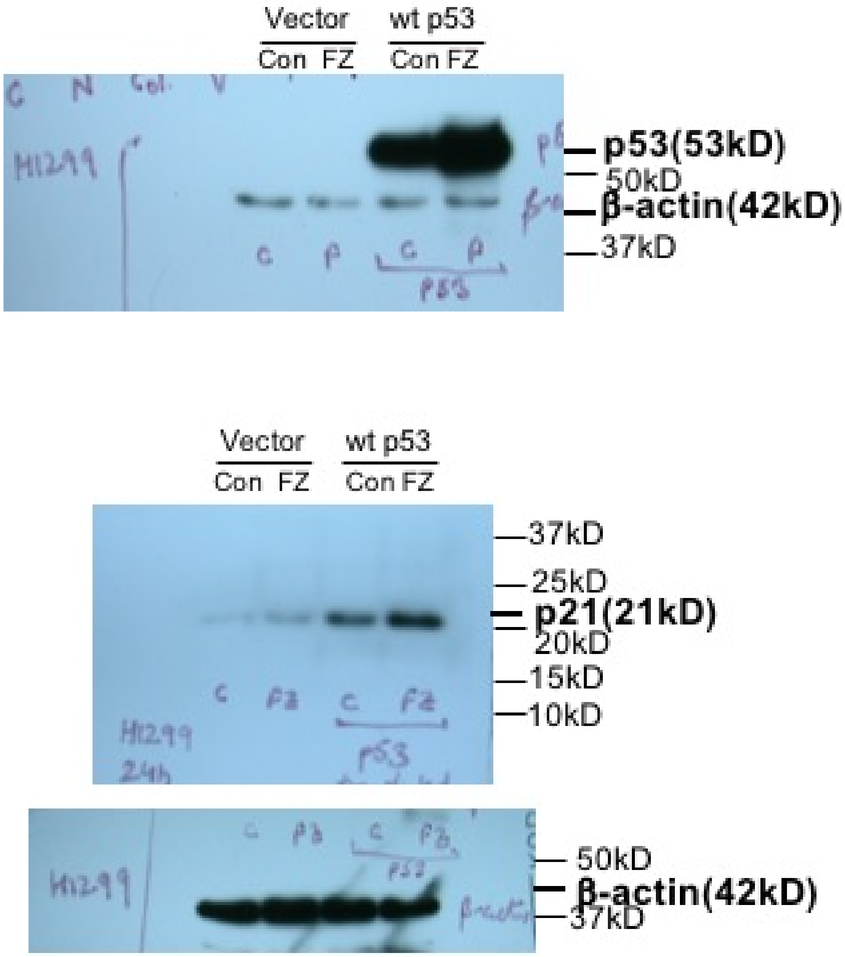
Vector
wt p53
Con FZ
Con FZ
p53(53kD)
50kD
β-actin(42kD)
37kD
Vector
wt p53
Con FZ
Con FZ
37kD
25kD
p21(21kD)
20kD
15kD
10kD
50kD
β-actin(42kD)
37kD

**Fig.7b**

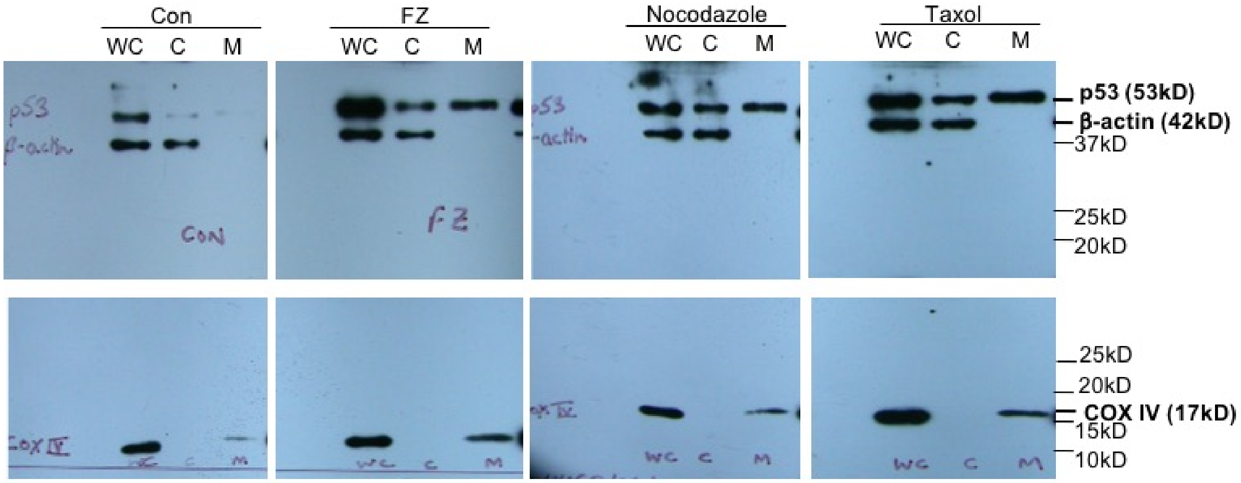
Con
WC C M
FZ
WC C M
Nocodazole
WC C M
Taxol
WC C M
p53 (53kD)
β-actin (42kD)
37kD
25kD
20kD
25kD
20kD
COX IV (17kD)
15kD
10kD
